# A novel PET tracer ^18^F-deoxy-thiamine: synthesis, metabolic kinetics, and evaluation on cerebral thiamine metabolism status

**DOI:** 10.1186/s13550-020-00710-5

**Published:** 2020-10-20

**Authors:** Changpeng Wang, Siwei Zhang, Yuefei Zou, Hongzhao Ma, Donglang Jiang, Lei Sheng, Shaoming Sang, Lirong Jin, Yihui Guan, Yuan Gui, Zhihong Xu, Chunjiu Zhong

**Affiliations:** 1 Department of Neurology, Zhongshan Hospital, State Key Laboratory of Medical Neurobiology and Institute of Brain Science, Fudan University, Shanghai, 200032 China; 2 Jiangsu Huayi Technology Co., Ltd., Jiangsu, 215519 China; 3 PET Center, Huashan Hospital, Fudan University, Shanghai, 200235 China; 4 Department of Clinical Pharmacology, Zhongshan Hospital, Fudan University, Shanghai, 200032 China

**Keywords:** Thiamine, ^18^F-deoxy-thiamine, Tracer, Positron emission tomography

## Abstract

**Background:**

Some neuropsychological diseases are associated with abnormal thiamine metabolism, including Korsakoff–Wernicke syndrome and Alzheimer’s disease. However, in vivo detection of the status of brain thiamine metabolism is still unavailable and needs to be developed.

**Methods:**

A novel PET tracer of ^18^F-deoxy-thiamine was synthesized using an automated module via a two-step route. The main quality control parameters, such as specific activity and radiochemical purity, were evaluated by high-performance liquid chromatography (HPLC). Radiochemical concentration was determined by radioactivity calibrator. Metabolic kinetics and the level of ^18^F-deoxy-thiamine in brains of mice and marmosets were studied by micro-positron emission tomography/computed tomography (PET/CT). In vivo stability, renal excretion rate, and biodistribution of ^18^F-deoxy-thiamine in the mice were assayed using HPLC and γ-counter, respectively. Also, the correlation between the retention of cerebral ^18^F-deoxy-thiamine in 60 min after injection as represented by the area under the curve (AUC) and blood thiamine levels was investigated.

**Results:**

The ^18^F-deoxy-thiamine was stable both in vitro and in vivo. The uptake and clearance of ^18^F-deoxy-thiamine were quick in the mice. It reached the max standard uptake value (SUVmax) of 4.61 ± 0.53 in the liver within 1 min, 18.67 ± 7.04 in the kidney within half a minute. The SUV dropped to 0.72 ± 0.05 and 0.77 ± 0.35 after 60 min of injection in the liver and kidney, respectively. After injection, kidney, liver, and pancreas exhibited high accumulation level of ^18^F-deoxy-thiamine, while brain, muscle, fat, and gonad showed low accumulation concentration, consistent with previous reports on thiamine distribution in mice. Within 90 min after injection, the level of ^18^F-deoxy-thiamine in the brain of C57BL/6 mice with thiamine deficiency (TD) was 1.9 times higher than that in control mice, and was 3.1 times higher in ICR mice with TD than that in control mice. The AUC of the tracer in the brain of marmosets within 60 min was 29.33 ± 5.15 and negatively correlated with blood thiamine diphosphate levels (*r* = − 0.985, *p* = 0.015).

**Conclusion:**

The ^18^F-deoxy-thiamine meets the requirements for ideal PET tracer for in vivo detecting the status of cerebral thiamine metabolism.

## Introduction

Thiamine, also named vitamin B_1_, is an essential nutrient that can be acquired only via diet. After being absorbed from gastrointestinal tract, thiamine is delivered to all organs and tissues and converted into bioactive thiamine diphosphate (TDP) [1]. Thiamine deficiency (TD) is associated with some neuropsychological diseases, such as Wernicke–Korsakoff syndrome, Alzheimer’s disease (AD), beriberi, Leigh syndrome, and so forth, and results in lactic acidosis, mitochondrial dysfunction, and energy deficits in brain, muscle, and heart, causing a broad range of clinical manifestations, such as anorexia, agitation, diminished tendon reflexes, ataxia, disturbance of consciousness, muscle pain, and heart failure. [2–4].

Glucose is the predominant substrate of brain energy metabolism and plays a pivotal role in maintaining cerebral function [5], which makes the brain vulnerable to glucose dysmetabolism. TDP is the common coenzyme of the three key enzymes in glucose catabolism: pyruvate dehydrogenase and α-ketoglutarate dehydrogenase in the Krebs cycle that is responsible for producing ATP in mitochondria, and transketolase in pentose phosphate pathway that generates antioxidants and the substrates of biosynthesizing DNA, RNA, and fatty acid in cytosol [6]. Therefore, the brain also is susceptible to TD by disturbing glucose metabolism [2, 7–12]

**Fig. 1:**
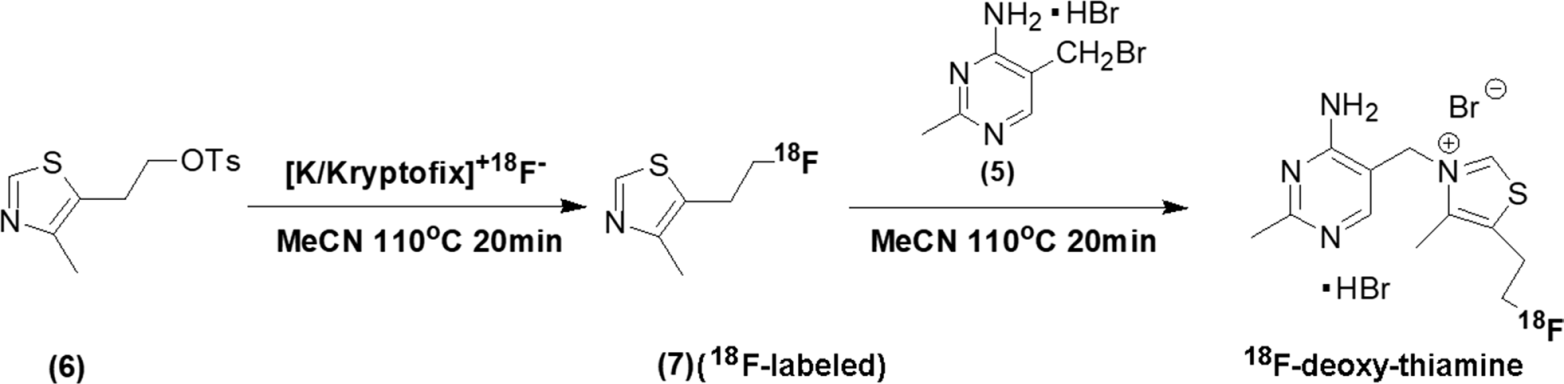
The two-step synthesis of ^18^F-deoxy-thiamine

However, in vivo detection of cerebral thiamine metabolism status is still unavailable. In this study, a novel radiotracer of positron emission topography (PET)—^18^F-deoxy-thiamine—was designed and synthesized (Fig. 1). The metabolic kinetics and retention of ^18^F-deoxy-thiamine in the brains of animals were studied.

## Materials and methods

### General information

All reagents were purchased from TCI Development Co., Ltd (Shanghai) and J & K Scientific (Beijing, China) unless otherwise indicated. Thin layer chromatography (TLC) was performed with silica gel layers, and compounds were visualized under UV light. The ^1^H NMR (300 MHz) spectra of all compounds were acquired on an Advance (Bruker) spectrometer. Chemical shifts (*δ*) for the proton resonance were reported in parts per million (ppm) downfield from TMS (*δ* = 0). The identification and purity of precursors as well as cold standard sample (^19^F-deoxy-thiamine) were determined by a LC–MS instrument (1200/6120, Agilent Technologies Inc.) with a C_18_ column (4.6 * 150 mm, 5 uM; VP-ODS, Shimadzu) at 0.5 ml per minute (ml/min) flow rate. The mobile phase consisted of 60% methanol and 40% H_2_O containing millesimal formic acid.

^18^F ions were obtained from a cyclotron (Cyclone 18 Twin, IBA, Belgium), situated at the Molecular Imaging institute, Jiangsu Huayi Technology Co., Ltd., by the nuclear reaction [^18^O(p,n)^18^F]. For automatic synthesis of ^18^F-deoxy-thiamine, we used a remote-controlled radiolabeling module (RNplus, Synthra) with slight modifications and created the sequence program, based on manual trials. The scheme of modified RNplus module is presented in Additional file 1: Figure 1. We used six reagent supply vials (A1–A5, B1) at the upper part and two reaction vials (vial I and vial II) at the bottom part.

Analytical HPLC (1260, Agilent Technologies Inc.) with the same type of column mentioned above was employed for ^18^F-deoxy-thiamine characterization and identification. The signal acquisition system consisted of a UV detector (254 nm) and a radio-detector (1IINaI/PMT, Lablogic, USA) in series. The flow rate was 0.8 ml/min, and the mobile phase consisted of methanol and H_2_O containing 0.05% triethylamine and 50 mM ammonium acetate. The percentage of methanol/H_2_O changed with running time: 0–15 min (mins), 15%/85%; 15–25 min, methanol increasing to 100% while H_2_O decreasing to 0%; 25–30 min, methanol 100%.

A micro-PET/CT equipment (Inveon; Siemens Co., USA) was used for detecting the levels of ^18^F-deoxy-thiamine in the organs of mice and marmosets. During micro-PET/CT scanning, the body temperature of animals was maintained at 37 °C using a heat pad.

C57BL/6 and Institute of Cancer Research (ICR) mice (obtained from the SLAC Laboratory Animal Company, China) were housed in a controlled environment at temperature of 20–26 °C and humidity of 40–70% with free access to food and water. The marmosets were supplied by Jiuting Non-human Primate Facility, Chinese Academy of Sciences, Shanghai. Four marmosets aged 3.1–10.8 years old were employed for micro-PET/CT scanning (M1: 3.1 year, female; M2: 3.5 year, male; M3: 5.4 year, female; M4: 10.8 year, male). At the day of experiment, the marmosets were fetched from the facility and were sent back when the scanning finished.

All animal care and experimental procedures were carried out according to the guidelines of the Animal Care Committee of Fudan University. This study was approved by Medical Experimental Animal Administrative Committee of Fudan University, and the committee on medical ethics of Zhongshan Hospital, Fudan University.

### Synthesis of cold standard sample of ^18^F-deoxy-thiamine as well as precursors (5) and (6).

We synthesized cold standard sample of ^18^F-deoxy-thiamine as well as precursors (5) and (6) according to the work of Cline JK, et al. [13], with minor modifications (Additional file 2: Figure 2). The purity of the cold standard sample is > 99.9%, and the purity of two precursors > 99%, identified via HNMR and LC–MS (Additional files 4, 5, 6, 7, 8, 9: Figure 3B–G). The details of the synthetic route were described in the Additional file 14: supplementary text.

### Automated radiosynthesis of ^18^F-deoxy-thiamine

We adopted a two-step synthesis route (Fig. 1). The scheme of automated synthesis was shown in Additional file 1: Figure 1. Reagents were added into supply vials as follows: A1: 1.1 ml eluent (3.08 mg KHCO_3_, 11 mg Kryptofix 2.2.2, 0.88 mL MeCN, 0.22 mL H_2_O); A2: 1 ml MeCN; A3: 5 mg precursor (**6**) in 0.5 ml MeCN; A4: 1 ml MeCN; A5: 0.5 ml MeCN; B1: 0.5 ml H_2_O. 5 mg precursor (**5**) powder was also added into reaction vial II beforehand. When the first step synthesis finished, the intermediate product [^18^F]-compound (**7**) was transferred from reaction vial I to reaction vial II via distillation. The whole automated synthesis duration was 100 min. The details of synthesis and purification were described in the Additional file 14: supplementary text.

### Characterization and quality control of ^18^F-deoxy-thiamine

Radiochemical yield (RCY, decay-correction to the end of bombardment) and radiochemical concentration (RCC) were measured by the radioactivity calibrator (CRC-55tR, CAPINTEC, INC., USA). ^18^F-deoxy-thiamine was identified by co-injecting final product with cold standard sample into analytical HPLC. Radiochemical purity (RCP) and specific radioactivity (SA) were calculated by means of the area under curve (AUC) of radio-signals and UV-signals of final product in analytical HPLC, respectively. Bacteria and endotoxin detections were carried out by means of anaerobic/aerobic bacteria media and Limulus reagent gel methods, respectively, according to Chinese Pharmacopoeia.

### In vitro stability

^18^F-deoxy-thiamine solution was stored at room temperature (RT) and injected into analytical HPLC for evaluating RCP and peak shape at 0 h (h), 2 h, 4 h, 6 h, 8 h, and 10 h, respectively, after synthesized.

### Thiamine deficiency mouse model

Eight-week-old male C57BL/6 and ICR mice were randomly divided into two groups: TD mouse models (*n* = 2 for C57BL/6, one died due to anesthesia during micro-PET/CT scanning; *n* = 3 for ICR) were established by feeding thiamine-deprived diet (Trophic Animal Feed High-tech Co., Ltd., China). Control mice (*n* = 3 for each strain) were fed the general diet. Twenty-eight days later, all mice received PET/CT scanning.

### Micro-PET/CT imaging

Micro-PET/CT imaging using ^18^F-deoxy-thiamine as the tracer was performed in the mice with TD and control mice, as well as in marmosets. The animals were anesthetized by inhaling 1.5–2% of isoflurane in air (1.5 L/min) and received CT scan for acquiring structure image and attenuation correction data. Then, the mice were injected with 7.4–14.8 MBq of ^18^F-deoxy-thiamine in 0.1 ml volume (diluted by normal saline) through the tail vein. Brain PET imaging was immediately performed and dynamically acquired for 90 min with an energy window of 350–650 KeV and a time window of 3.438 ns. A total of 35 frames were setup: 20 f, 3 s; 4 f, 60 s; 5 f, 300 s; 6 f, 600 s. Dynamic images were reconstructed by OSEM3D/SP-MAP algorithm with two iterations. After scanning, the mice were sacrificed.

The marmosets were injected with 46.3–74.0 MBq of ^18^F-deoxy-thiamine in 0.5–0.8 ml volume through the femoral vein. Brain PET imaging was immediately scanned and dynamically acquired for 60 min. A total of 18 frames were setup: 6 f, 10 s; 4 f, 60 s; 5 f, 300 s; 6 f, 600 s. The blood samples of the marmosets were taken from femoral vein for measuring the levels of thiamine, TMP, and TDP. The other conditions for marmosets were the same as that for mice.

Regions of interest (ROIs) were drawn manually over the whole brain (for mice and marmosets) and in the left ventricular cavity (for marmosets) based on the PET/CT co-registered images using IRW 4.2 software (Siemens Medical Solutions USA, Inc.). Radioactivity was expressed as standard uptake value (SUV): (ROI radioactivity/ROI volume)/(injected radioactivity/gram of body weight). The time-activity curve (TAC) and AUC (SUV * mins) were also calculated.

The TACs of the marmosets blood (Radioactivity was expressed as SUV) were taken as input functions (IF) [14–16] for fitting Patlak plots [17, 18], in order to analyze transfer constants (K_i_) of brains in marmosets (IRW 4.2 software). The details of Patlak model were described in the Additional file 14: supplemental text.

### Measurement of thiamine, TMP, and TDP in whole blood samples of marmosets

Thiamine, TMP, and TDP levels in whole blood samples were measured using HPLC, based on the established method [4] with slight modification. Briefly, blood samples were collected using heparin-anticoagulated tubes; 150 ul sample was vibrated for 30 s with equal volume of 5.2% perchloric acid (PCA) added dropwise for deproteinization. Then, the mixture was stored at − 80 °C until assay within 1 month. The mixture was centrifuged at 12,000 rpm for 8 min at 4 °C; the supernatant was pipetted. Thiamine, TMP, and TDP in supernatant were derivatized into thiochromes using potassium ferricyanide and analyzed by gradient elusion with C_18_ reversed-phase analytical column (250 * 4.6 mm). The derivatives were identified by HPLC fluoroscopy (1100, Agilent Technologies Inc., ex: 367 nm, em: 435 nm). The thiamine, TMP, and TDP levels were quantified using standard samples (Sigma-Aldrich, St. Louis, MO). The analyzers were blinded to samples information.

### Studies of pharmacokinetic and metabolic kinetics in liver and kidney of mice

Nine-week-old male ICR mice (*n* = 5) were dynamically scanned using micro-PET/CT for 60 min. A total of 18 frames were reconstructed: 6 f, 10 s; 4 f, 60 s; 5 f, 300 s; 6 f, 600 s. The scanning conditions were the same as those mentioned above. The ROIs of liver and renal parenchyma as well as left ventricular cavity were manually drawn.

For pharmacokinetic study, the TACs of the blood were fitted. The radioactivity was evaluated as %IA/g (the percentage of injected activity per gram of blood). Pharmacokinetics parameters were counted through the software PKSolver (version 2.0, China Pharmaceutical University) [19].

For metabolic kinetics study, SUV, TAC, AUC, maximum radioactivity (*C*_max_), and time to *C*_max_ (*T*_max_) were calculated. The TACs of the blood (Radioactivity expressed as SUV) were taken as IFs for fitting Logan plots [20, 21], in order to analyze the distribution volumes (*V*_D_) of ^18^F-deoxy-thiamine in liver and kidney, respectively (IRW 4.2 software). The details of Logan model were described in the Additional file 14: supplemental text.

### Biodistribution study

The biodistribution of ^18^F-deoxy-thiamine was studied in ICR mice (*n* = 36 in total; 18 males, 9-week-old, 33.5 ± 4.0 g; 18 females, 7-week-old, 27.7 ± 5.0 g). For each mouse, 0.1 ml of ^18^F-deoxy-thiamine solution (37 MBq/ml) was injected into the tail vein under isoflurane anesthesia. The mice were sacrificed at 5 min, 10 min, 30 min, 1 h, 2 hs, and 4 hs after injection (3 males and 3 females for each time point). The tissues of heart, liver, spleen, lung, kidney, stomach, duodenum, pancreas, femur, muscle (from thigh), artery blood, brain, fat, and gonad (ovary or testicle) were harvested, weighted, and measured for radioactivity by γ-counter. %IA/g was calculated referring to the counts of standard samples.

### In vivo stability and the renal excretion rate

After metabolic kinetics study (Heading 9, Materials and Methods section), the mice were stopped to exposure to isoflurane and woke up several mins later. The urine accumulated in the bladder of the mice during the whole anesthetization period would be excreted. Some mice were softly rubbed the lower bowel in order to promote urination as completely as possible. The duration from the injection of ^18^F-deoxy-thiamine to mouse urination was about 85 min. The urine samples from three ICR mice were collected using syringes and measured in the radioactivity calibrator. Then, 0.1 ml urine for each mouse was added in an Eppendorf tube, vibrated for 30 s with equal volume of PCA added dropwise for deproteinization. After centrifuging at 12,000 rpm for 8 min at 4 °C, the supernatant was filtered and analyzed using HPLC.

### Statistical analysis

For the continuous data, mean ± standard error of mean (SEM) was applied for statistical description. Student's *t* test was employed to compare the AUC values between TD mice and controls in ICR strain. The Pearson correlation was utilized to analyze the correlation between the cerebral accumulation of ^18^F-deoxy-thiamine and the levels of blood thiamine, TMP, and TDP in marmosets. Repeated measurement of ANOVA with Tukey’s post hoc was used to analyze the AUC values of ^18^F-deoxy-thiamine in brain, liver, and kidney in ICR mice. All statistical analyses were performed using SPSS (Statistical Package for the Social Sciences) software (version 22.0; SPSS Inc., Chicago IL).

## Results

### Characterization and quality control of ^18^F-deoxy-thiamine

**Fig. 2:**
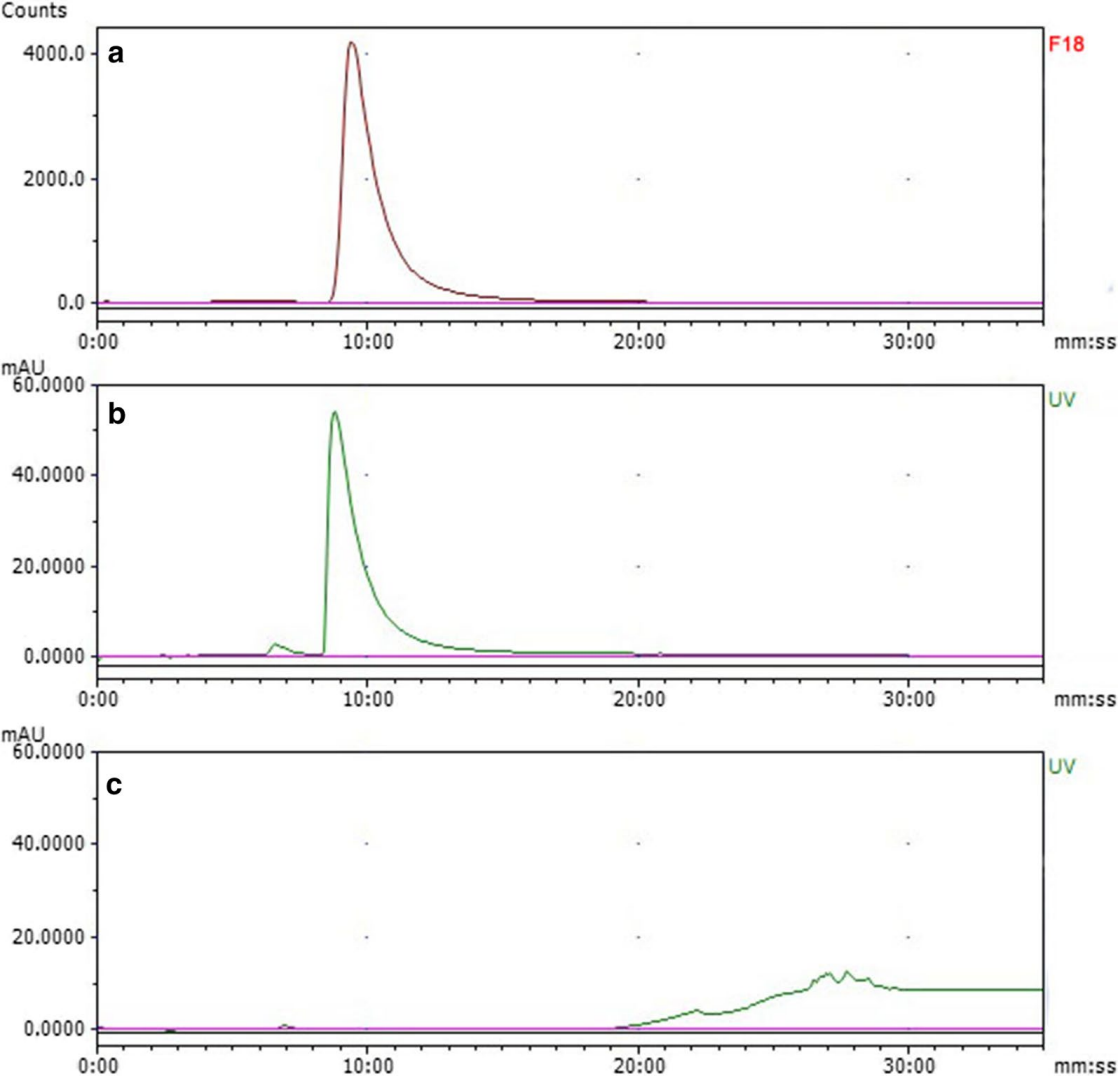
The identity of ^18^F-deoxy-thiamine and the measurements of radioactivity purity and specific activity. **a**, **b** Results of co-injection of ^18^F-deoxy-thiamine with cold standard sample. **a** was the radio-signal, and the retention time was 9 min 24 s; **b** was the UV-signal, and the retention time was 8 min 47 s. Radio-signal acquisition system was installed behind UV-signal acquisition system, and the time-lag was about half-minute at the flow rate of 0.8 ml/min. Note that the peak shapes of **a**, **b** were the same. **c** the UV signal of ^18^F-deoxy-thiamine. UV = 254 nm

The solution of ^18^F-deoxy-thiamine was clear and free of any particulate matter. All relevant items of characterization are shown in Table 1. The RCP and SA were 98.28 ± 0.39% and > 55.5 GBq/umol, respectively (Fig. 2). The RCY was 5.17 ± 1.04% (decay-corrected to the end of bombardment), and the RCC was 740–1110 MBq/ml. The bacteria and endotoxin tests were negative. For exploring the expiration time, we tested the stability in vitro. ^18^F-deoxy-thiamine final product was stable at RT, with the RCP > 95% 6 h, and > 93% 10 h after synthesis (Table 2).

**Table 1:** Characterization and quality control of ^18^F-deoxy-thiamine

Items	Results
Physical character	Clear and transparent, no macroscopic impurity
pH value	4.5
Alcohols	0%
RCP (*n* = 3)	98.28 ± 0.39%
SA	> 55.5 GBq/umol
RCY (*n* = 3)^a^	5.17 ± 1.04%
RCC (*n* = 3)	986.67 ± 123.33 MBq/ml
Bacteria test	Negative
Endotoxin test	Negative

^a^Decay-corrected to the end of bombardment

**Table 2:** In vitro stability test of ^18^F-deoxy-thiamine

Time after synthesis (h)	RCP (%)
0	97.68
2	96.89
4	96.11
6	95.03
8	94.01
10	93.38

### Evaluation of cerebral thiamine metabolism status

#### C57BL/6 mice

**Fig. 3:**
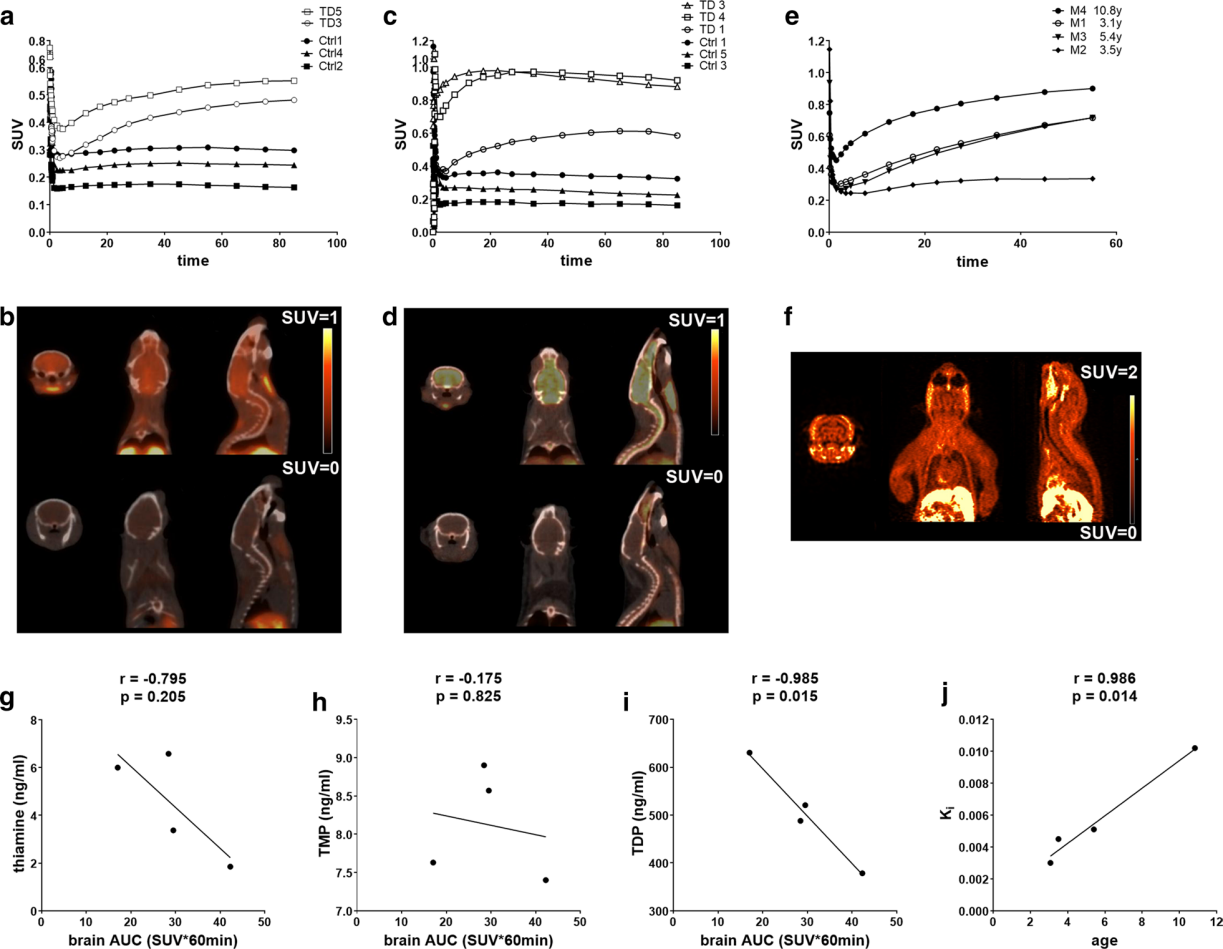
The evaluation of cerebral thiamine metabolism status in mice and marmosets. **a**, **b** C57BL/6 mice. A: the cerebral TACs of ^18^F-deoxy-thiamine in 90 min. The average AUC value in two mice with TD was 1.9 times higher than that in control mice (38.73 ± 3.74 vs. 20.38 ± 3.23). **b** The representative PET/CT images in TD and control groups (TD 5 and Ctrl 2, both at 50–60 min). **c**, **d** ICR mice. **c** The cerebral TACs of ^18^F-deoxy-thiamine in 90 min. The AUC values in mice with TD were significantly higher than those in controls (68.28 ± 10.74 vs. 21.88 ± 4.25, *P* = 0.016). **d** The representative PET/CT images in TD and control mice (TD 4 and Ctrl 1, 50–60 min). **e**, **j** Marmosets. **e** The cerebral TACs of ^18^F-deoxy-thiamine in 60 min. **f** The representative PET/CT images (M4, 30–60 min). **g**–**i** The correlations between cerebral AUC values of ^18^F-deoxy-thiamine within 60 min and blood levels of thiamine, TMP, and TDP. **j** The correlation between cerebral transfer constant *K*_i_ and age

The cerebral retention of ^18^F-deoxy-thiamine was increasing and approached to *C*_max_ at the terminal time of 90 min in the two mice with TD. The SUVs_max_ of the last frame (80–90 min) were 0.48 (TD 3 mouse), and 0.55 (TD 5 mouse). The AUC values within 90 min were 34.99 (TD 3 mouse) and 42.46 (TD 5 mouse). In contrast, the cerebral retention was stable within 90 min in the three control mice; the SUVs_max_ were about 0.17 (Ctrl 2 mouse), 0.25 (Ctrl 4 mouse), and 0.3 (Ctrl 1 mouse). The AUC values within 90 min were 14.52 (Ctrl 2 mouse), 20.96 (Ctrl 4 mouse), and 25.67 (Ctrl 1 mouse) (Fig. 3a, b). The mean of AUC values in TD mice was 1.9 times higher than that in control mice (38.73 ± 3.74 vs. 20.38 ± 3.23).

#### ICR mice

The values of cerebral SUVs_max_ in three mice with TD were 0.98 at 28th frame (TD 3 mouse, 20–25 min), 0.97 at 30th frame (TD 4 mouse, 30–40 min), and 0.61 at the 33th frame (TD1 mouse, 60–70 min), and then, the SUVs declined slowly. The AUC values within 90 min were 79.28 (TD 3 mouse), 78.75 (TD 4 mouse), and 46.81 (TD 1 mouse), respectively. The SUVs_max_ of ICR control mice were 0.36 (Ctrl 1 mouse), 0.27 (Ctrl 5 mouse), and 0.18 (Ctrl 3 mouse) within 90 min, respectively. The TACs in control mice were stable, similar to that in the C57BL/6 strain mice. The AUC values within 90 min were 29.58 (Ctrl 1 mouse), 21.14 (Ctrl 5 mouse) and 14.92 (Ctrl 3 mouse), respectively (Fig. 3c, d). The mean of AUC values in TD mice was 3.1 times higher than that in controls (68.28 ± 10.74 vs. 21.88 ± 4.25, *P* = 0.016).

#### Marmosets

The retention of ^18^F-deoxy-thiamine in the cerebrum of three marmosets had been increasing within 60 min (M1, M3, M4) and reached plateau at 16^th^ frame (30–40 min) in M2. The values of SUV_max_ were between 0.33 and 0.90, and the AUC values within 60 min were between 17.07 and 42.28 (29.33 ± 5.15; Fig. 3e, f). There was a significantly negative correlation between cerebral AUC values within 60 min and TDP levels in whole blood samples (*r* = − 0.985, *p* = 0.015, Fig. 3i). No significant correlation was found between the AUC values of ^18^F-deoxy-thiamine in cerebrum and blood thiamine and TMP levels (Fig. 3g, h). In order to quantitatively evaluate the uptake of ^18^F-deoxy-thiamine in the cerebrum of marmosets, the Patlak model was utilized to analyze the blood-to-brain transfer rate represented by constant K_i_ based on the characteristics of metabolic kinetics in the brain. The regression plots were fitted automatically by software from 20 to 60 min. *K*_i_ was between 0.0030 and 0.0102 ml/g/min, and significantly correlated with age (*r* = 0.986, *P* = 0.014, Fig. 3j, see the fitted Patlak plots in Additional file 10: Figure 4).

### Pharmacokinetic study and metabolic kinetics study in liver and kidney of mice

**Fig. 4:**
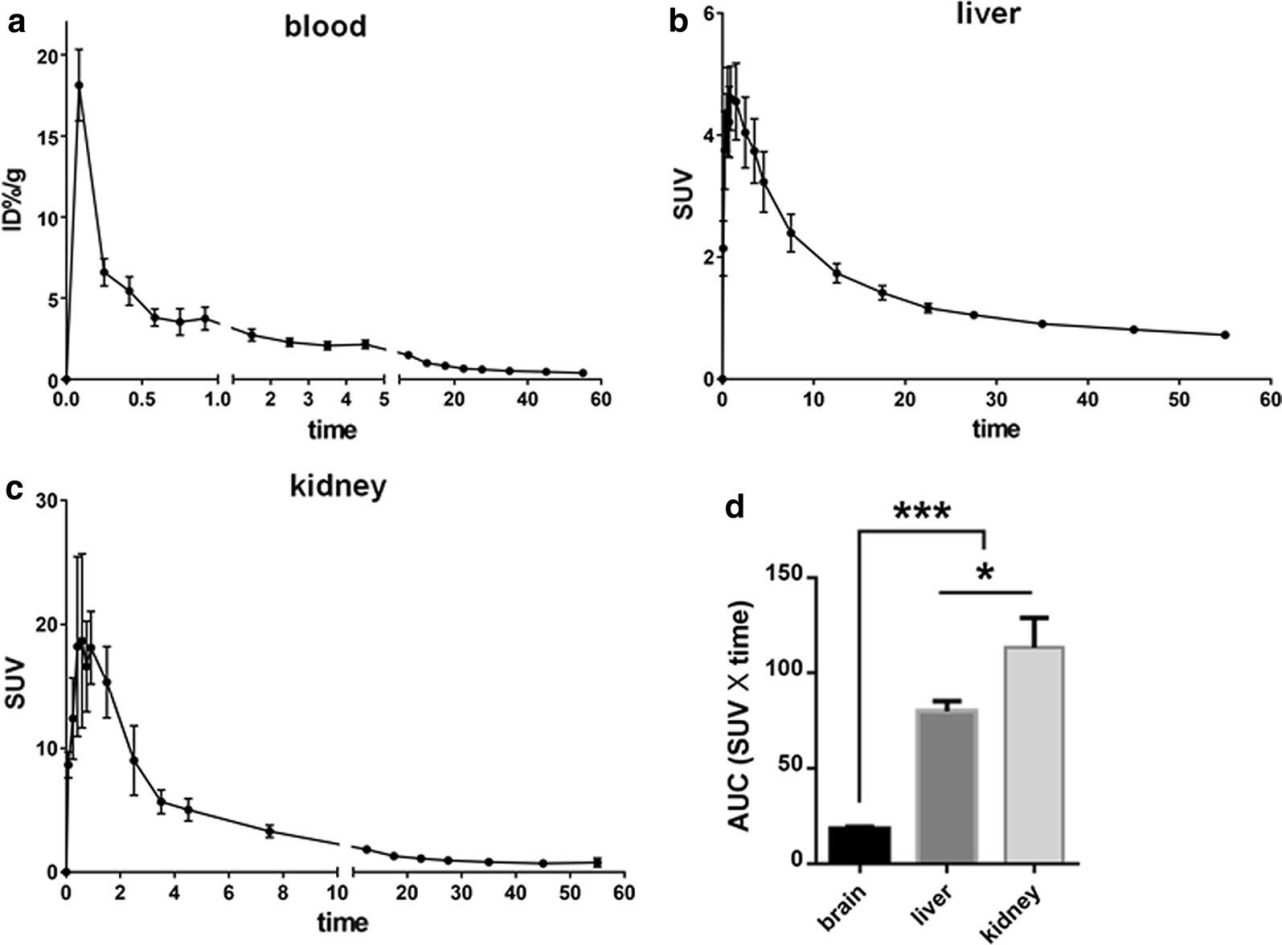
Studies of pharmacokinetics and metabolic kinetics of ^18^F-deoxy-thiamine in ICR mice. **a** The TAC of ^18^F-deoxy-thiamine in blood (radioactivity expressed as IA%/g). **b**, **c** The TACs of ^18^F-deoxy-thiamine in liver and kidney, respectively (radioactivity expressed as SUV). D. Comparisons of AUC values within 60 min between brain, liver, and kidney. Note that the scale of X axes in **a**, **c** was changed properly. *P < 0.05. ***P < 0.001. Repeated measurement of ANOVA with Tukey’s post hoc, *n* = 5 for each graph

Table 3 shows the pharmacokinetic parameters. Figure 4a shows the TAC of ^18^F-deoxy-thiamine (radioactivity as IA%/g) within 60 min in the ICR mice blood represented by the ROI in left ventricle. The pharmacokinetic profiles of ^18^F-deoxy-thiamine fitted a two-compartment open model. The value of AUC_0–60 min_ was 37.313, accounting for 99.77% of the value of AUC_0–inf_ (37.399), which implies that the 60 min observation be sufficient to depict the pharmacokinetic characters. The T_max_ was in the first frame (0–10 s) and the *C*_max_ was 18.12 ± 2.20 IA%/g, respectively. The half-life of distribution (*t*_1/2α_) and half-life of elimination (*t*_1/2β_) were 0.082 and 6.379 min. The volume of distribution (*V*_D_1) and clearance rate (CL1) of central compartment were 3.058 g and 2.674 g/min. The *V*_D_2 and CL2 of peripheral compartment were 19.308 g and 20.162 g/min. These results revealed that ^18^F-deoxy-thiamine could be absorbed and eliminated rapidly.

**Table 3:** Pharmacokinetic parameters of ^18^F-deoxy-thiamine in ICR mice (*n* = 5)

Parameters (units)	Values
*T*_max_ (s)	0–10
*C*_max_ (IA%/g)	18.12 ± 2.20^a^
*V*_D_1 (g)	3.058
CL1 (g/min)	2.674
*V*_D_2 (g)	19.308
CL2 (g/min)	20.162
*t*_1/2_(*α*) (min)	0.082
*t*_1/2_(*β*) (min)	6.379
AUC_0–60 min_ (%IA/g * min)	37.313
AUC_0–inf_ (%IA/g * min)	37.399
*k*_10_ (min^−1^)^b^	0.874
*k*_12_ (min^−1^)^b^	6.593
*k*_21_ (min^−1^)^b^	1.044

^a^Data were expressed as mean ± SEM

^b^*K*_10_, elimination rate constant; *K*_12_, elimination rate constant from central compartment to peripheral compartment; *K*_21_, elimination rate constant from peripheral compartment to central compartment

**Fig. 5:**
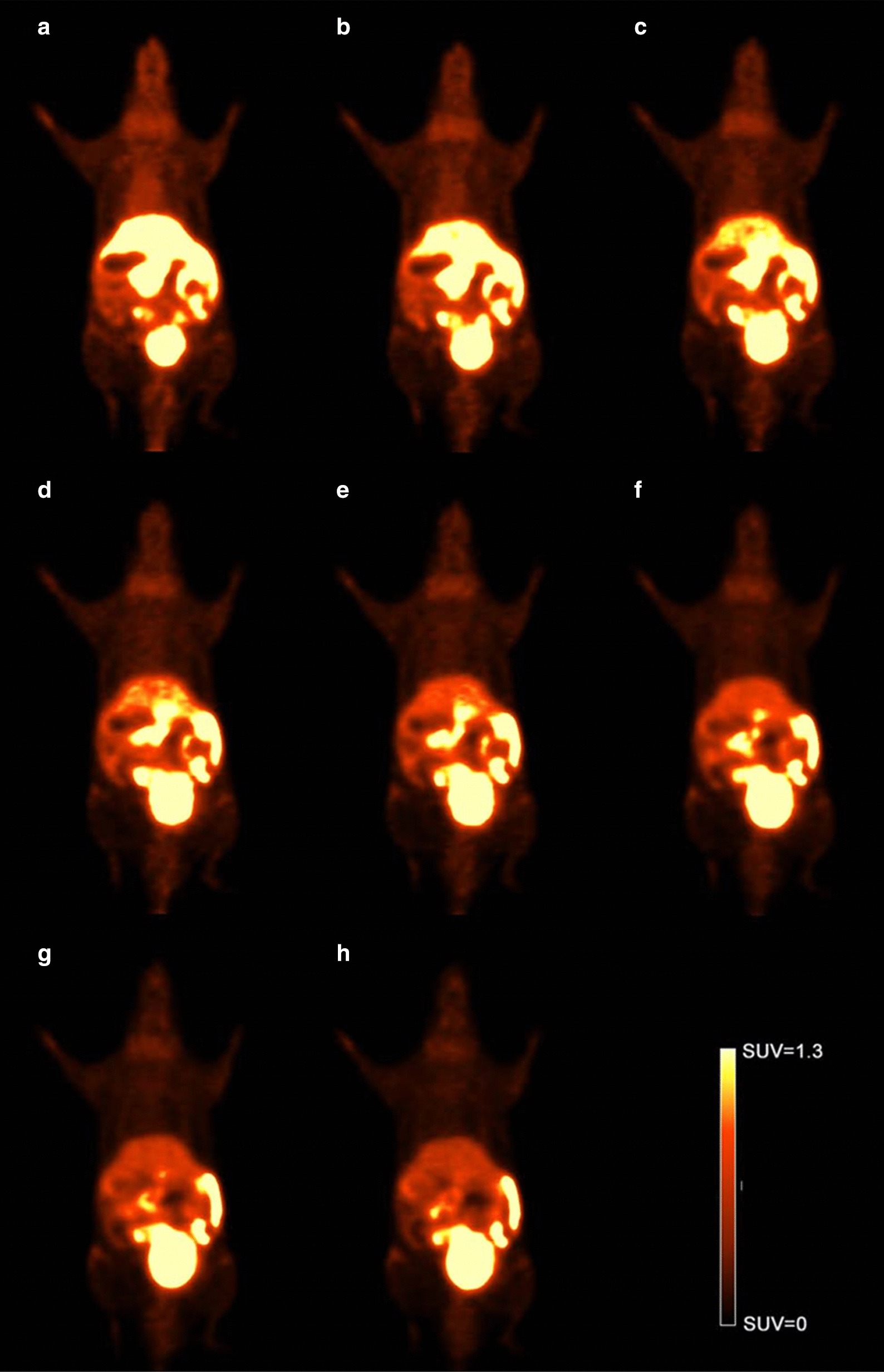
Dynamic micro-PET/CT Scanning within 60 min. The representative results from one ICR mouse. **a** The 11th frame (5–10 min). **b** The 12th frame (10–15 min). **c** The 13th frame (15–20 min). **d** The 14th frame (20–25 min). **e** The 15th frame (25–30 min). **f** The 16th frame (30–40 min). **g** The 17th frame (40–50 min). **h** The 18th frame (50–60 min)

The representative images of dynamic micro-PET/CT whole-body scanning of ICR mice within 60 min are shown in Fig. 5. Initially, ^18^F-deoxy-thiamine distributed mainly into liver and bladder. Then, the radioactivities in the liver quickly declined, while that in bladder was increasing over time. These results demonstrated that the uptake and elimination of ^18^F-deoxy-thiamine in vivo were fast and mainly occurred in liver and kidney.

The *T*_max_ in liver was approximately 1 min post-injection. The value of SUV_max_ was 4.61 ± 0.53. Then, the SUV dropped fast to 3.23 ± 0.50 at approximately 5 min. The radioactivity continued to decline afterward. The SUV was 0.72 ± 0.05 at the terminal time (60 min). The AUC value within 60 min was 79.94 ± 5.43 (Fig. 4b). The *T*_max_ in the kidney was approximately 30 s, and the SUV_max_ was 18.67 ± 7.04. The SUV dropped fast to 3.29 ± 0.50 in 10 min and continued to drop slowly. At the terminal time (60 min), the SUV dropped to 0.77 ± 0.35. The AUC value was 113.4 ± 15.56 (Fig. 4c). The AUC values of liver and kidney within 60 min were significantly higher than that of whole brain (*P* < 0.001). Also, the AUC values in kidney were significantly higher than that in liver (*P* < 0.05, Fig. 4d).

Logan plot was applied to analyze the distribution volume (*V*_D_) of liver and kidney based on the characteristics of metabolic kinetics. The regression plots were fitted automatically by software from 10 to 60 min, and *V*_D_ were 4.573 ± 0.34 ml/g in the liver and 6.17 ± 0.88 ml/g in the kidney (Additional file 11, 12: Figure 5A–E, 6A–E). The results indicated that the “steady-state” of uptake/clearance was reached in the 10th min, and the mean uptake amount of ^18^F-deoxy-thiamine per gram tissue was equivalent to the amount contained in 4.573 ml (for liver) or 6.17 ml (for kidney) of blood.

### In vivo stability and renal excretion rate

**Fig. 6:**
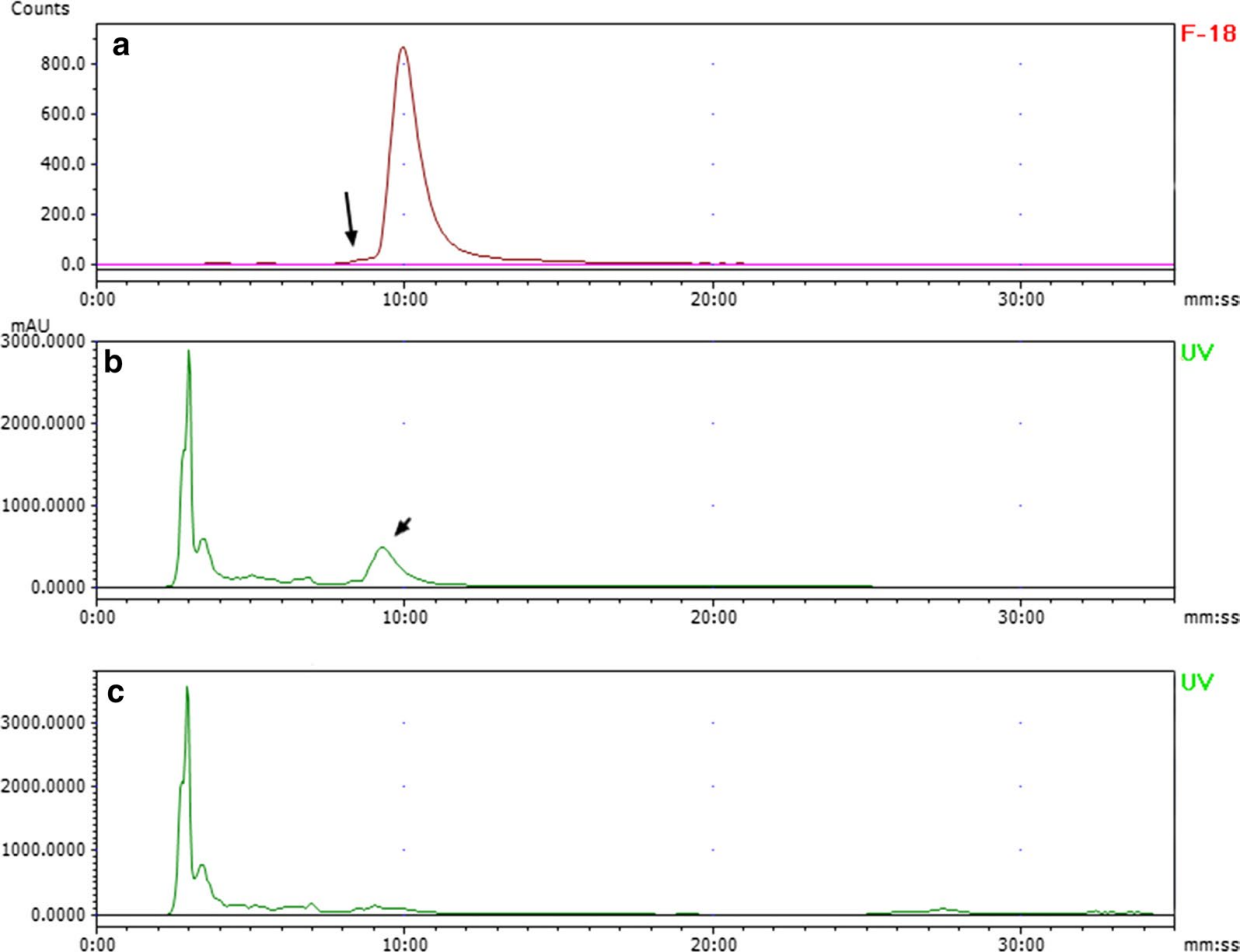
The representative results of in vivo stability study on ^18^F-deoxy-thiamine. **a**, **b** HPLC results for the mixture of mouse urine extracts and cold standard sample of ^18^F-deoxy-thiamine. **a** The radio-signal, and the retention time of ^18^F-deoxy-thiamine was 9 min 54 s. Note that the unknown metabolite(s) was ahead of ^18^F-deoxy-thiamine (arrow). **b** The UV-signal, and the retention time of cold standard sample was 9 min 14 s (arrowhead). The UV-signal acquisition system was ahead of radio-signal, and the time-lag was about half-minute. **c** The UV signal of analyzing only the mouse urine extracts. UV = 254 nm. The peak around 3 min in **b**, **c** was the metabolic compounds excreted through kidney into urine. Although the urine was treated by deproteinization, centrifugation, and filtration, many in-vivo metabolic compounds could not be separated and removed

Since the -OH group of thiamine is replaced by -^18^F, ^18^F-deoxy-thiamine cannot enter the known metabolic pathways of thiamine till ^18^F decays back to ^18^O^−^ [22, 23]. The radioactivity in the collected urine was 34.16 ± 3.84% of total injected radioactivity measured by radioactivity calibrator (decay-correction), and no correlation was found between the excreted radioactivity in urine and the injected radioactivity (Additional file 14: Table 1). The RCP of renal excreted ^18^F-deoxy-thiamine was 94.53 ± 0.81% determined by HPLC. In addition, an unknown trace substance (3.30 ± 0.60% in RCP) was found ahead of ^18^F-deoxy-thiamine, which could not be ^18^F^−^ ions according to its retention time and shape (Fig. 6). The percentage of intact tracer was 96.63%. These results indicated that about one-third of ^18^F-deoxy-thiamine was excreted by kidney and that ^18^F-deoxy-thiamine was stable within 85 min in vivo.

### Biodistribution study

**Fig. 7:**
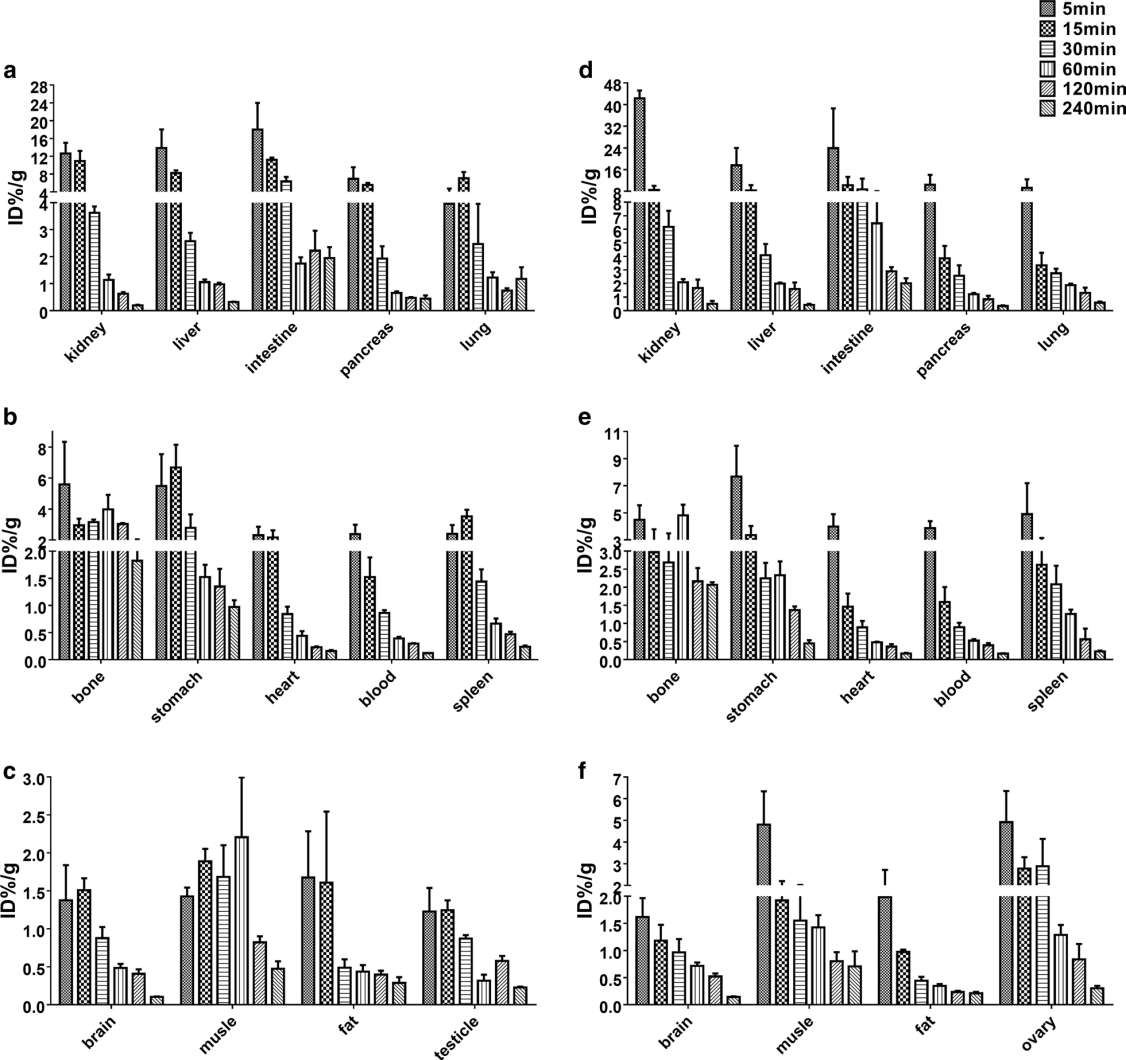
Biodistribution of ^18^F-deoxy-thiamine in ICR mice. **a**–**c** Male. **d**–**f** Female. Radioactivity accumulation was expressed as %IA/g at 5, 15, 30, 60, 120, 240 min after tail-vein injection of 3.7 MBq ^18^F-deoxy-thiamine in 0.1 ml volume. *n* = 3 for each organ or tissue at each time point, except for kidney, lung at 5 min of male, and kidney, liver, duodenum, lung, ovary at 5 min of female (*n* = 2)

As a derivative of an essential vitamin, ^18^F-deoxy-thiamine distributed widely in vivo. In most of the organs and tissues of ICR mice, the *C*_max_ of ^18^F-deoxy-thiamine achieved between 5 and 15 min, then ^18^F-deoxy-thiamine was cleared rapidly, and the accumulation was very low in these organs and tissues till 4 h after injection (Fig. 7, Additional file 14: Table 2). The muscular radioactivity in female ICR mice began to decline 5 min after injection, differentiating from that in male ICR mice, in which the radioactivity increased within 60 min, then declined rapidly.

Consistent with the results of PET/CT scanning, the biodistributions of liver and kidney were higher than that in the other organs investigated. The biodistributions of ^18^F-deoxy-thiamine in brain, fat, and gonad were less than those in other organs or tissues observed in this study. Interestingly, the level of ^18^F-deoxy-thiamine in the ovary was higher than that in the testicle, indicating the difference in ^18^F-deoxy-thiamine metabolism between male and female gonads.

## Discussion

The levels of thiamine and its phosphate esters decline in the brains of mice with TD [24]. Our current study showed the retention of ^18^F-deoxy-thiamine was higher in the cerebra of two strains mice with TD as compared with that in control mice (Fig. 3a–d). The results indicate that the enhanced accumulation of ^18^F-deoxy-thiamine in the brain could reflect the status of cerebral thiamine deficiency of the mice. It may be due to the fact that brain elevates the uptake efficiency of thiamine from periphery blood in order to meet the demands of high rate of glucose metabolism as well as other thiamine-dependent physiological activities under the condition of thiamine deficiency. TDP accounts for 90% of total thiamine in the body [25]. We observed that the level of ^18^F-deoxy-thiamine in the brain significantly negatively correlated with blood TDP levels in marmosets (Fig. 3i). In addition, the blood-to-brain transfer rate as represented by *K*_i_ was significantly positively correlated with the age of marmosets (Fig. 3j), suggesting that the reserve of thiamine in marmosets declines with age. The result is consistent with our previous observation in the non-demented elderly [26]. Of course, this result should be further validated by expanding the sample size in future studies.

The previous study has showed that TDP reduction contributes to cerebral glucose hypometabolism of AD, and the concentration of blood TDP was positively correlated with the level of cerebral glucose metabolism in AD patients [24]. Cerebral glucose metabolism is extremely high [27]. Under normal circumstances, the brain glucose concentration is kept constant at the millimole concentration and cerebral metabolic rate of glucose (CMR_Glu_) reaches 0.2–0.3 umol/g/min [28]. It may lead to the possibility that slight alteration of brain glucose metabolism under some pathological conditions is difficult to be detected by PET with [^18^F]-fluorodeoxyglucose (FDG-PET). In this study, the SUVs of ^18^F-deoxy-thiamine in the brains of control mice and marmosets were less than 0.4 and 0.9, respectively (Fig. 3a, c, e). It is much lower than that of FDG in the range of 1–2 in the brains of mice [29]. The results imply that PET with ^18^F-deoxy-thiamine may be more sensitive than FDG-PET for evaluation of mild brain hypometabolism status. The advantages and disadvantages of these two methods for detecting brain metabolic status should be further investigated in future studies.

In both genders of ICR mice, kidney, liver, and pancreas exhibit high accumulation level of ^18^F-deoxy-thiamine, while brain, muscle, fat, and gonad show low accumulation concentration (Fig. 7; Additional file 14: Table 2, 3). These results are consistent with previous reports on the biodistributions of thiamine and its phosphate esters [30–32]. Besides, we found that 3.37% of ^18^F-deoxy-thiamine in urine was metabolized to more polar compounds after 85 min in vivo process (Fig. 6). To our knowledge, the –OH group is the active site for thiamine converting into bioactive TDP in vivo [22, 23]. Thus, before –^18^F decays back to stable isotope –^18^O, ^18^F-deoxy-thiamine could not enter the known metabolic routes. The presence of this 3.37% compounds implied other unknown metabolic routes might exist. ^18^F-deoxy-thiamine could help for further exploration on the metabolism and biodistribution of thiamine.

Stability is important for PET tracer. ^18^F-deoxy-thiamine was stable at RT. The RCP was > 95% 6 h, and > 93% 10 h after synthesis, which means no significant radiolysis effect exists in vitro (Table 2) [33]. In addition, no ^18^F-deflourination of ^18^F-deoxy-thiamine in vivo was observed based on the phenomenon that the accumulation of radioactivity signal in bone was decreasing over time [34] (Fig. 7b, e). Also, the prototype form of ^18^F-deoxy-thiamine excreted in urine reached 96.63% within 85 min after vein injection (Fig. 6). These results demonstrated that ^18^F-deoxy-thiamine was highly stable both in vitro and in vivo.

Pharmacokinetic and metabolic kinetics studies as well as biodistribution study indicated that the uptake and clearance of ^18^F-deoxy-thiamine in vivo were fast, which is another important requirement. The *t*_1/2β_ was 6.379 min; CL_1_ and CL_2_ were 2.674 g/min and 20.162 g/min, respectively (Table 3). The values of *C*_max_ in various tissues and organs of ICR mice reached within 15 min (except for muscle of males, reached within 1 h), and the accumulation of ^18^F-deoxy-thiamine was very low in these tissues and organs till 4 h after injection (Fig. 7, Additional file 14: Table 2 and 3). Besides, at least one-third of ^18^F-deoxy-thiamine was excreted through kidney 85 min after injection. (A small amount of urine may be retained in bladder and not be collected.) See Additional file 14: supplementary Table 1.

Graphical analysis technique has been extensively applied in the analyses of nuclear medicine imaging data. It is especially suitable for pharmacokinetic studies of novel tracers before the compartmental models are fully described, because it is independent of any specific model configuration [21]. We analyzed the important kinetic parameters *K*_i_ and *V*_D_ in the brain of marmosets and in the liver and kidney of ICR mice employing Patlak and Logan plots, respectively, based on the characteristics of TACs in these organs (Figs. 3e, 4b, c). The *V*_D_ in the kidney and liver of ICR mice were 6.17 ± 0.88 ml/g and 4.573 ± 0.34 ml/g, respectively. The results showed that the uptake and clearance of ^18^F-deoxy-thiamine in vivo were fast.

Thiamine consists of a pyrimidine ring with an electronegative amidogen and a thiazole ring. The structural complexity determines the difficulty of artificially synthesizing and modifying thiamine. Since the first synthesis route was reported in 1937 [13], only a few studies on thiamine synthesis and modification have been published [35–37]. Recently, Doi et al. [37] synthesized radio-labeled thiamine with ^11^C and conducted heart imaging study in rats [38]. Although ^11^C-thiamine possesses the same molecular structure as thiamine itself, the short half-life of ^11^C (20.4 min) limits its application. Also, ^11^C-thiamine would be phosphorylated to ^11^C-TDP fast via the –OH group in vivo [38], which complicates the interpretation of the radio-signal. Here, ^18^F-deoxy-thiamine was successfully synthesized by a two-step route, in which –OH group of thiamine replaced by –^18^F (Fig. 1). Although the RCY was not high enough, the RCP, SA, and RCC were high (Table 1). The bacteria and endotoxin tests were negative. These results indicated that ^18^F-deoxy-thiamine was safe and suitable for in vivo studies.

The most difficult part in our two-step route is the isolation and purification of the intermediate product ^18^F-compound (**7**). Though we have tried various solid phase extractions as well as preparative HPLC, either the isolation failed or the process became too complicated to be automated. The molecule of 4-methyl-5(beta-hydroxyethyl)-thiazole is solid, and its boiling point is 135 °C under vacuum [39]. Once –OH is changed to –F, however, this compound becomes oil and volatile at RT. We speculated that it was because the H bond connecting H and N was broken (Additional file 13: Figure 7). By distillation, we successfully isolated ^18^F-compound (**7**) and realized the automation of the radiosynthesis route.

## Conclusion

In this study, we synthesized a novel PET tracer of ^18^F-deoxy-thiamine and established its automated synthesis route. Further, ^18^F-deoxy-thiamine was stable in vitro and in vivo, and possessed ideal characteristics of metabolic kinetics. The PET with ^18^F-deoxy-thiamine could evaluate the status of cerebral thiamine metabolism and might be more suitable for evaluating cerebral energy metabolism than FDG-PET due to the low abundance of cerebral thiamine metabolism. However, the sample size of marmosets in this study was not large enough, and we lack of TD model of marmosets to further investigate thiamine metabolism in non-human primates. This study laid the foundation for further studies on diseases related to thiamine dysmetabolism.

## Supplementary information

**Additional file 1: Figure 1.** The scheme of radio-synthesis using an automated module.

**Additional file 2: Figure 2.** The synthesis route of cold standard sample of ^18^F-deoxy-thiamine as well as precursors (5) and (6).

**Additional file 3: Figure 3A.** A: The TLC analyses results of intermediate products in the synthesis route of cold standard sample of 18F-deoxy-thiamine.

**Additional file 4: Figure 3B.** B: HNMR result of precursor 5.

**Additional file 5: Figure 3C.** C: LC-MS result of precursor 5, the purity has been highlighted.

**Additional file 6: Figure 3D.** D: HNMR result of precursor 6.

**Additional file 7: Figure 3E.** E: LC-MS result of precursor 6, the purity has been highlighted.

**Additional file 8: Figure 3F.** F: HNMR result of cold standard sample of 18F-deoxy-thiamine.

**Additional file 9: Figure 3G.** G: LC-MS result of cold standard sample of 18F-deoxy-thiamine, the purity has been highlighted.

**Additional file 10: Figure 4.** Blood-to-brain transfer rate constant K_i_ of ^18^F-deoxy-thiamine analyzed by Patlak plot in marmosets. A: M1, 3.1 years old. B: M2, 3.5 years old. C: M3, 5.4 years old. D: M4, 10.8 years old. The unit of Ki is ml/g/min.

**Additional file 11: Figure 5.** Distribution volume (V_D_) of ^18^F-deoxy-thiamine in liver analyzed by Logan plot in ICR mice. A–E: Each figure is for one of five ICR mice, respectively. The unit of V_D_ is ml/g.

**Additional file 12: Figure 6.** Distribution volume (V_D_) of ^18^F-deoxy-thiamine in kidney analyzed by Logan plot in ICR mice. A–E: Each figure is for one of five ICR mice, respectively. The unit of V_D_ is ml/g.

**Additional file 13: Figure 7.** The H bond connecting H and N of 4-methyl-5(beta-hydroxyethyl)-thiazole is broken.

**Additional file 14.**

## Data Availability

The datasets used in the current study are available from the corresponding author on reasonable request.

## Abbreviations

TDP: Thiamine diphosphate
TMP: Thiamine monophosphate
TD: Thiamine deficiency
PPP: Pentose phosphate pathway
TLC: Thin layer chromatography
ppm: Parts per million
ml/min: Ml per minute
RCY: Radiochemical yield
RCC: Radiochemical concentration
RCP: Radiochemical purity
SA: Specific radioactivity
AUC: Area under curve
RT: Room temperature
ROI: Region of interest
SUV: Standard uptake value
TAC: Time-activity curve
IF: Input function
*K*_i_: Transfer constants
PCA: Perchloric acid
VD: Distribution volume
SEM: Standard error of mean
CL: Clearance rate
