# Supplementary figures and images for: A novel PET tracer 18F-deoxy-thiamine: synthesis, metabolic kinetics, and evaluation on cerebral thiamine metabolism status
Source: EJNMMI Res. 2020 Oct 20;10:126. doi: 10.1186/s13550-020-00710-5 (PMC7575681; doi:10.1186/s13550-020-00710-5)

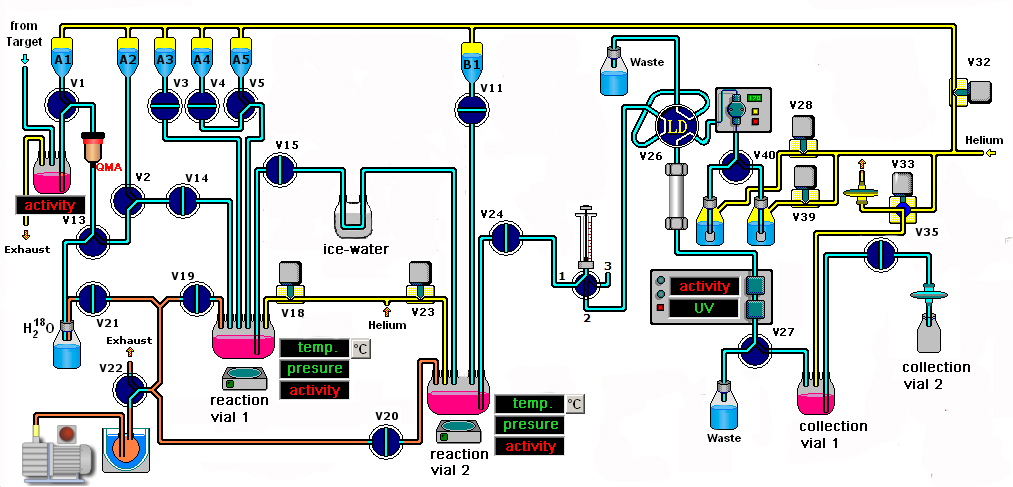

Supplement: Supplementary file 1 — Additional file 1: Figure 1. The scheme of radio-synthesis using an automated module. [file 13550_2020_710_MOESM1_ESM.tif]

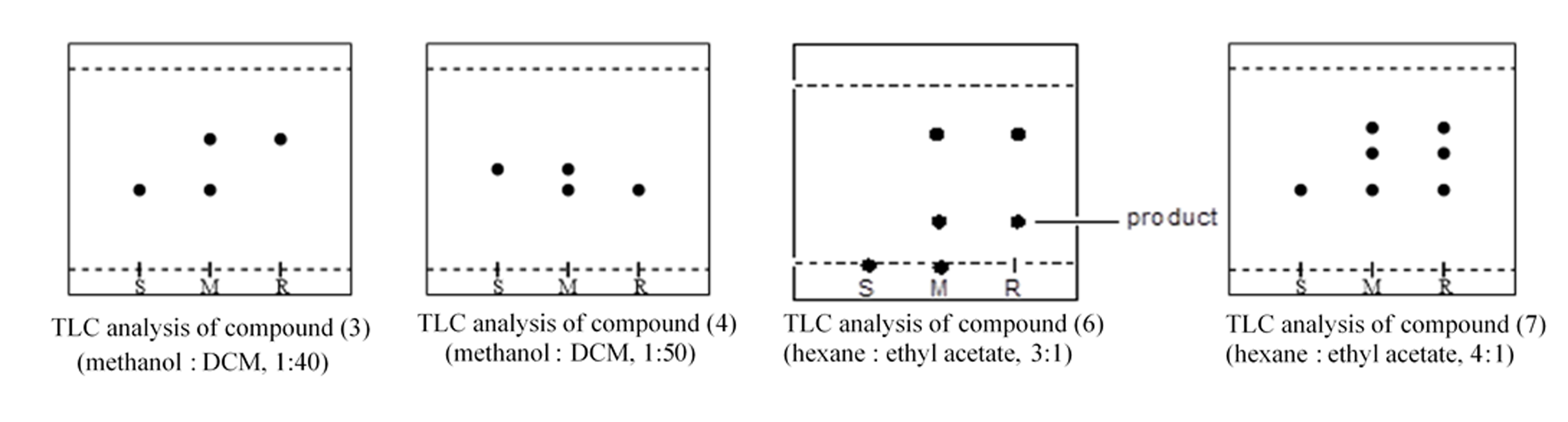

Supplement: Supplementary file 3 — Additional file 3: Figure 3A. A: The TLC analyses results of intermediate products in the synthesis route of cold standard sample of 18F-deoxy-thiamine. [file 13550_2020_710_MOESM3_ESM.jpg]

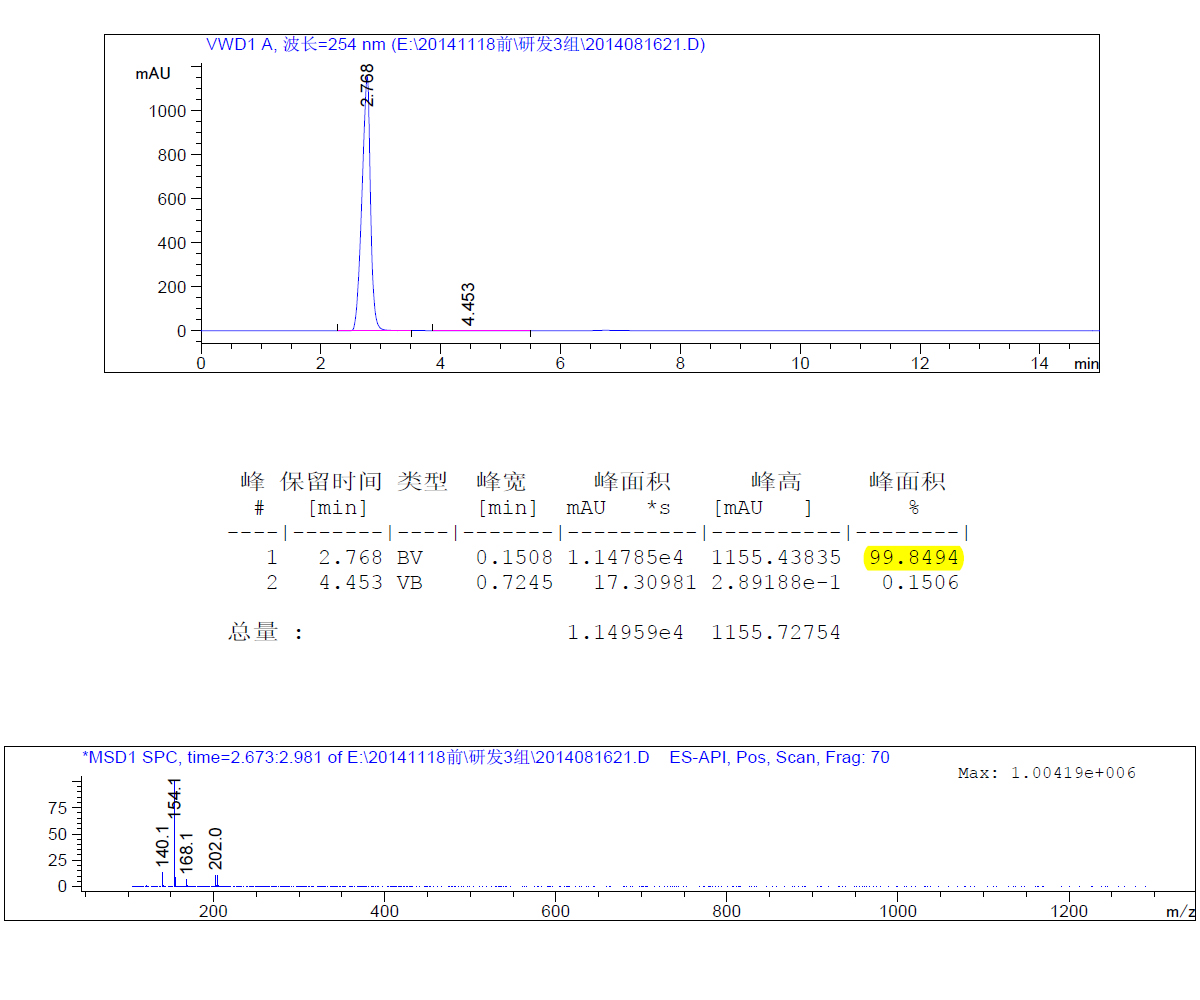

Supplement: Supplementary file 5 — Additional file 5: Figure 3C. C: LC-MS result of precursor 5, the purity has been highlighted. [file 13550_2020_710_MOESM5_ESM.jpg]

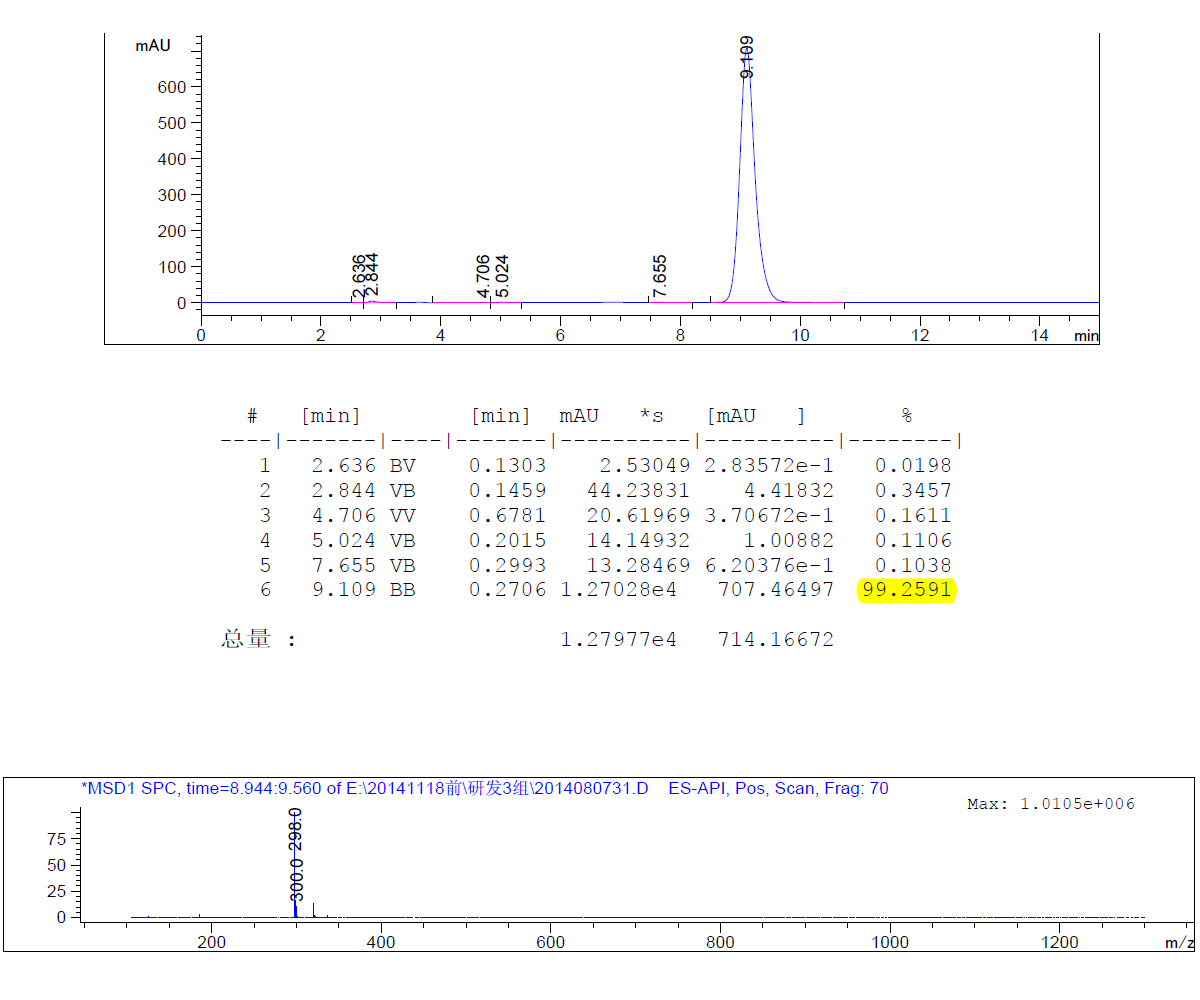

Supplement: Supplementary file 7 — Additional file 7: Figure 3E. E: LC-MS result of precursor 6, the purity has been highlighted. [file 13550_2020_710_MOESM7_ESM.jpg]

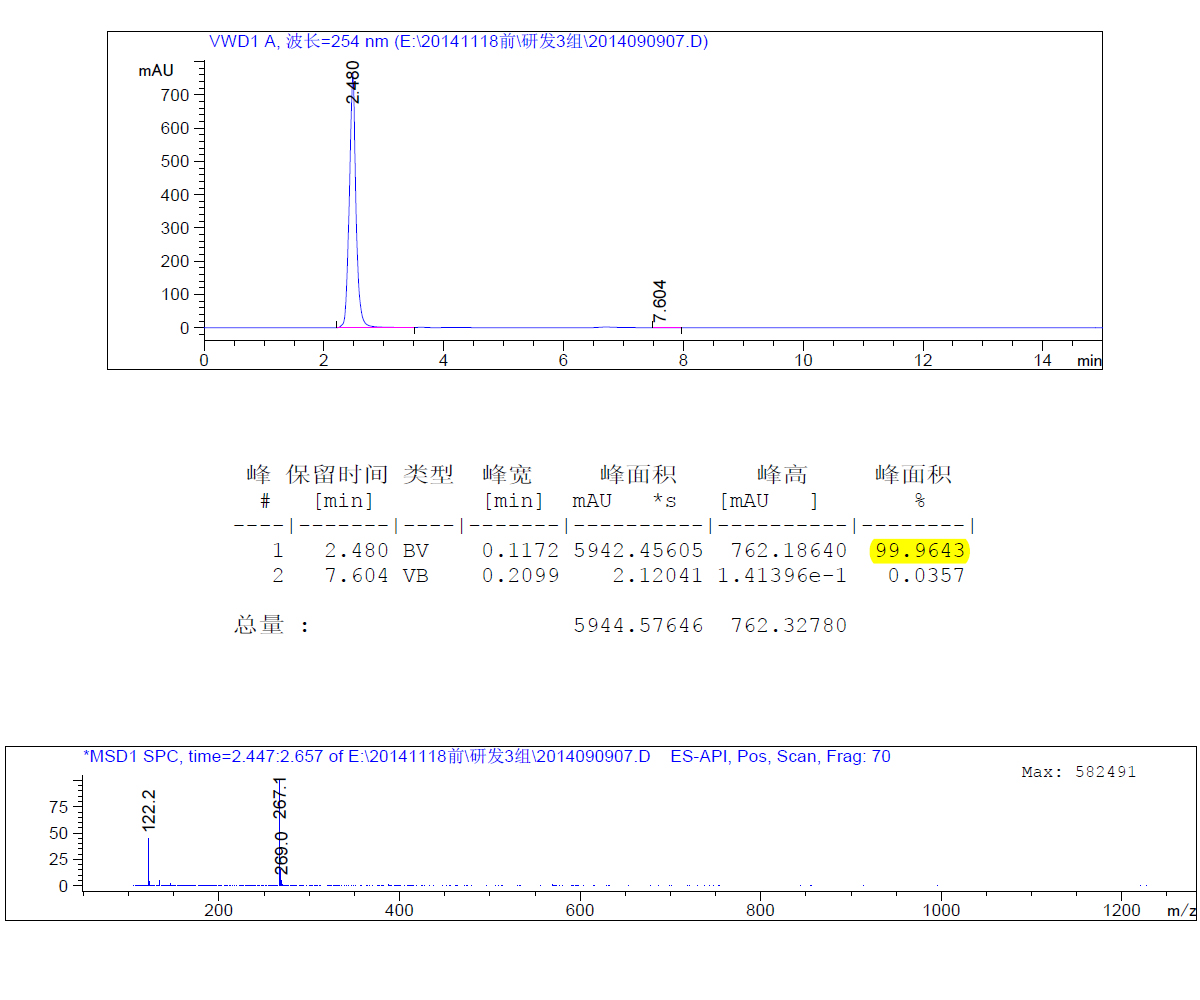

Supplement: Supplementary file 9 — Additional file 9: Figure 3G. G: LC-MS result of cold standard sample of 18F-deoxy-thiamine, the purity has been highlighted. [file 13550_2020_710_MOESM9_ESM.jpg]

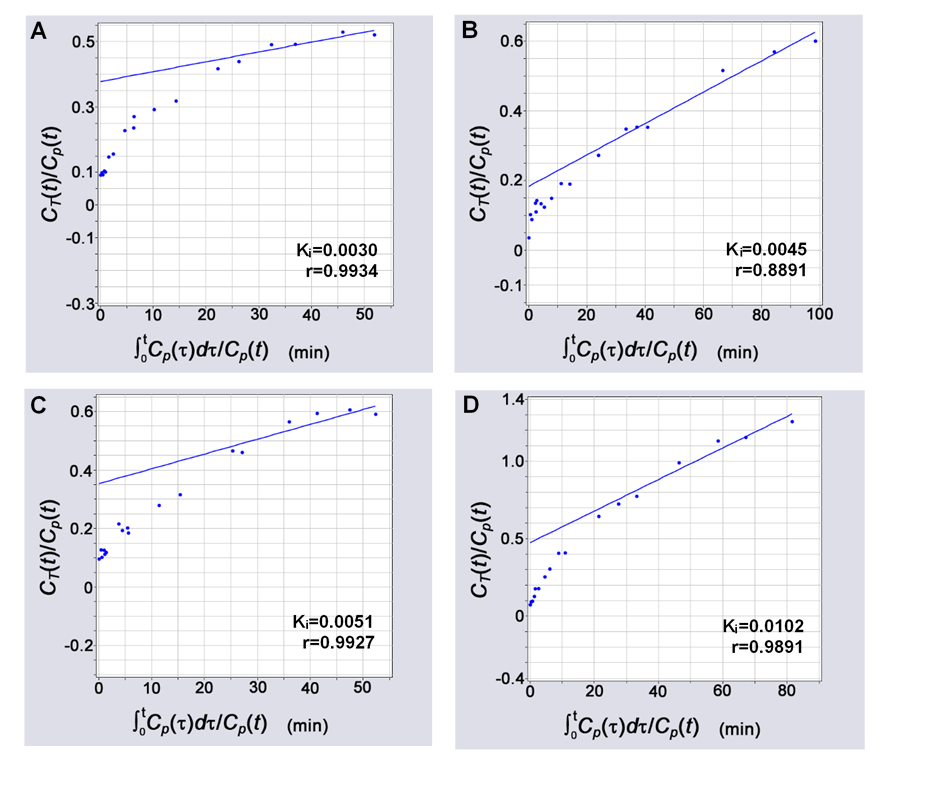

Supplement: Supplementary file 10 — Additional file 10: Figure 4. Blood-to-brain transfer rate constant Ki of 18F-deoxy-thiamine analyzed by Patlak plot in marmosets. A: M1, 3.1 years old. B: M2, 3.5 years old. C: M3, 5.4 years old. D: M4, 10.8 years old. The unit of Ki is ml/g/min. [file 13550_2020_710_MOESM10_ESM.tif]

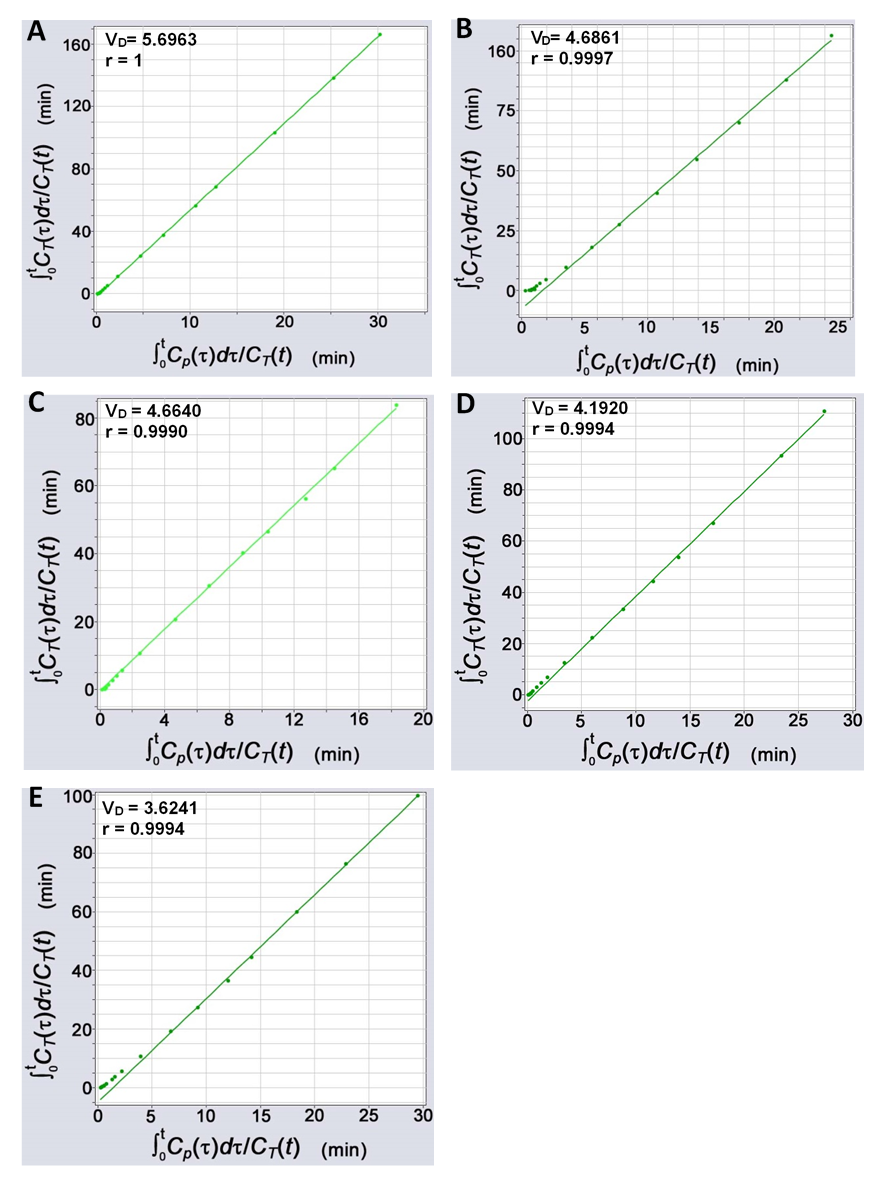

Supplement: Supplementary file 11 — Additional file 11: Figure 5. Distribution volume (VD) of 18F-deoxy-thiamine in liver analyzed by Logan plot in ICR mice. A–E: Each figure is for one of five ICR mice, respectively. The unit of VD is ml/g. [file 13550_2020_710_MOESM11_ESM.tif]

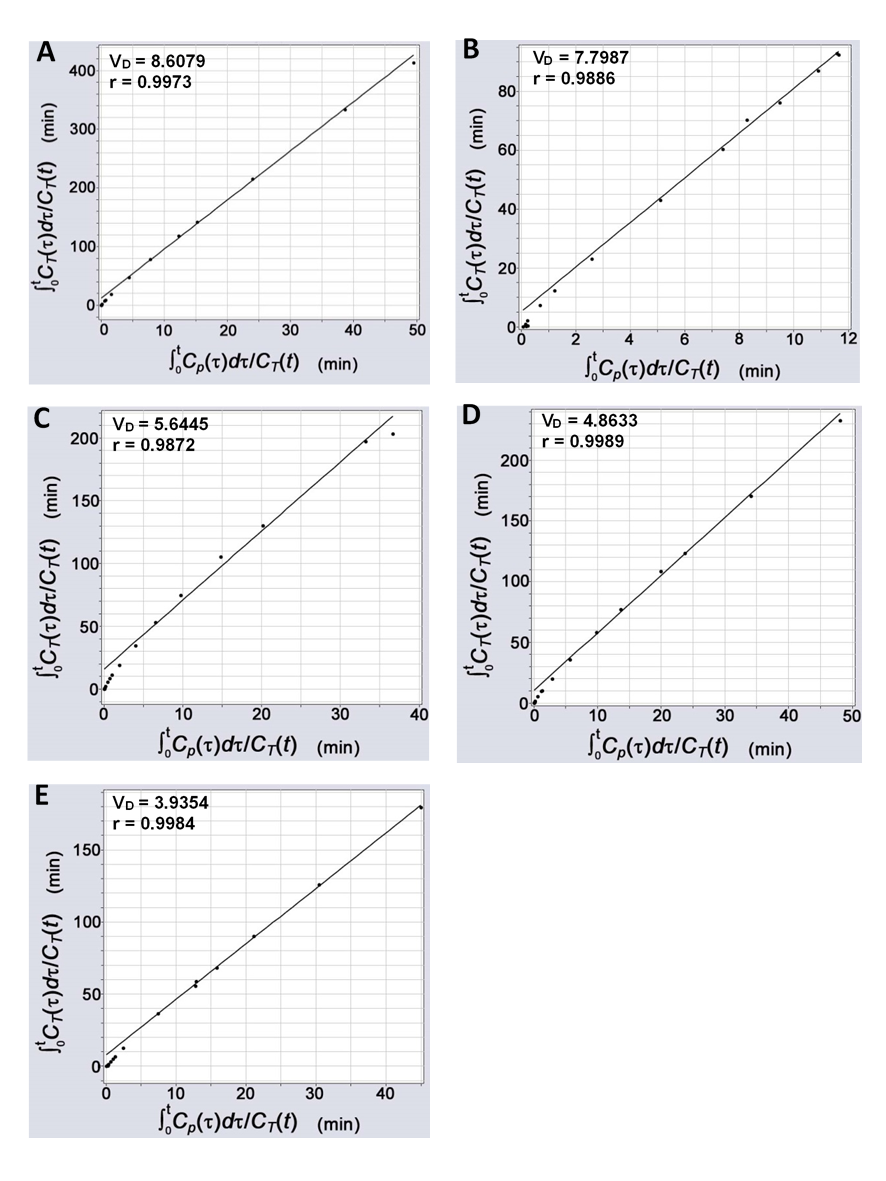

Supplement: Supplementary file 12 — Additional file 12: Figure 6. Distribution volume (VD) of 18F-deoxy-thiamine in kidney analyzed by Logan plot in ICR mice. A–E: Each figure is for one of five ICR mice, respectively. The unit of VD is ml/g. [file 13550_2020_710_MOESM12_ESM.tif]

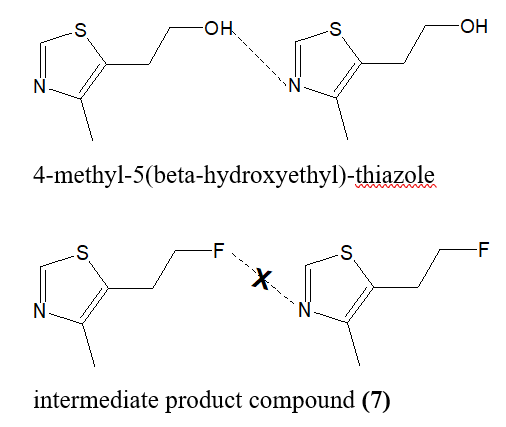

Supplement: Supplementary file 13 — Additional file 13: Figure 7. The H bond connecting H and N of 4-methyl-5(beta-hydroxyethyl)-thiazole is broken. [file 13550_2020_710_MOESM13_ESM.tif]
