# Supplementary material for: A novel PET tracer 18F-deoxy-thiamine: synthesis, metabolic kinetics, and evaluation on cerebral thiamine metabolism status
Source: EJNMMI Res. 2020 Oct 20;10:126. doi: 10.1186/s13550-020-00710-5 (PMC7575681; doi:10.1186/s13550-020-00710-5)
Supplement: Supplementary file 6 — Additional file 6: Figure 3D. D: HNMR result of precursor 6. [file 13550_2020_710_MOESM6_ESM.pdf]

MVBS

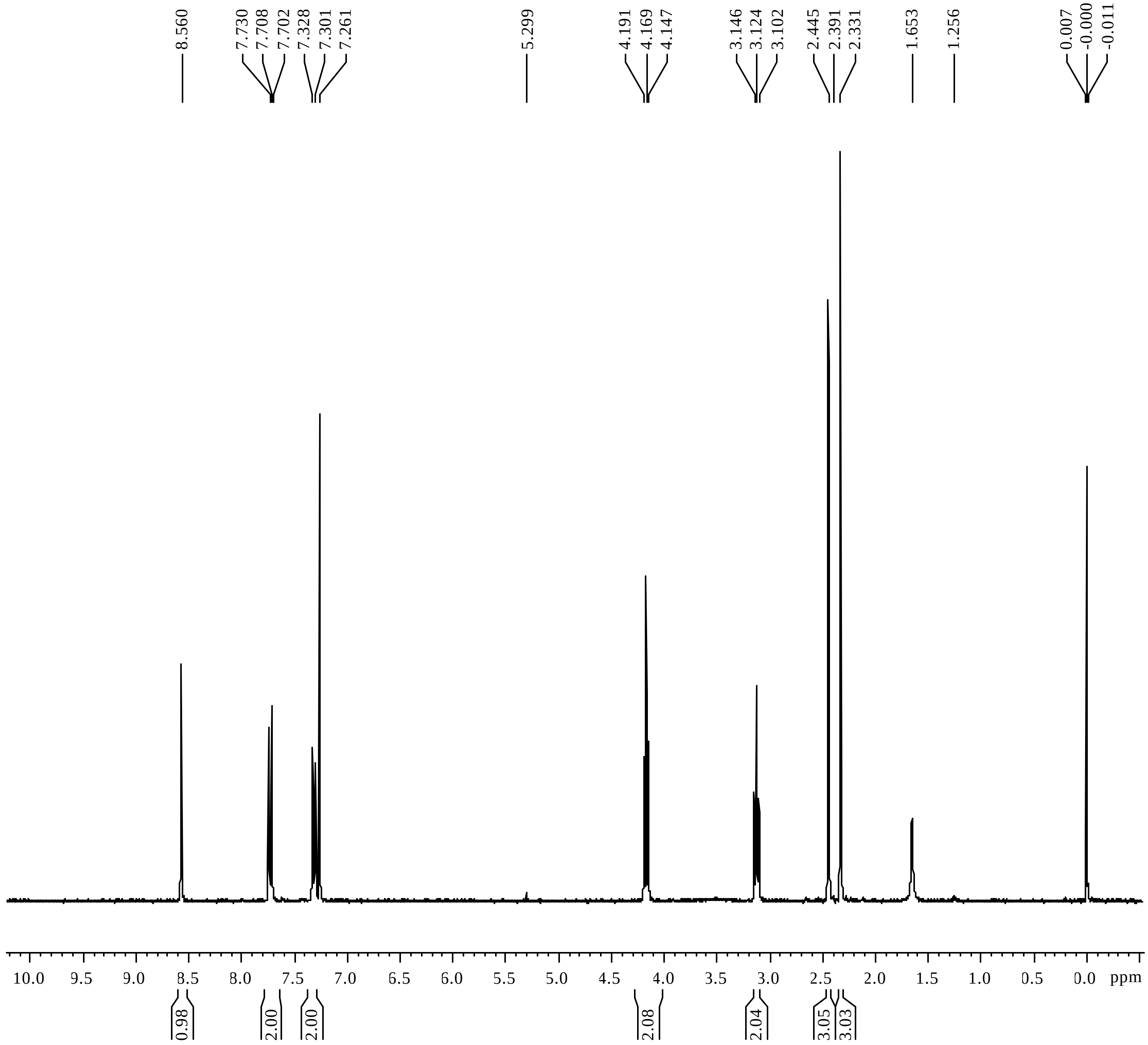

Current Data Parameters
NAME team 3
EXPNO 183
PROCNO 1

F2 - Acquisition Parameters
Date_ 20140728
Time 14.57
INSTRUM spect
PROBHD 5 mm PABBO BB-
PULPROG zg30
TD 32768
SOLVENT CDCl3
NS 8
DS 0
SWH 5411.255 Hz
FIDRES 0.165138 Hz
AQ 3.0278132 sec
RG 228.1
DW 92.400 usec
DE 6.00 usec
TE 298.3 K
D1 2.00000000 sec
TD0 1

======== CHANNEL f1 ========
NUC1 1H
P1 10.00 usec
PL1 -3.00 dB
SFO1 300.1320000 MHz

F2 - Processing parameters
SI 16384
SF 300.1300064 MHz
WDW EM
SSB 0
LB 0.00 Hz
GB 0
PC 1.00
