# Supplementary material for: A novel PET tracer 18F-deoxy-thiamine: synthesis, metabolic kinetics, and evaluation on cerebral thiamine metabolism status
Source: EJNMMI Res. 2020 Oct 20;10:126. doi: 10.1186/s13550-020-00710-5 (PMC7575681; doi:10.1186/s13550-020-00710-5)
Supplement: Supplementary file 14 — Additional file 14. [file 13550_2020_710_MOESM14_ESM.docx]

**1. Synthesis of cold standard sample of ^18^F-deoxy-thiamine as well as precursors (5) and (6).**

The synthesis route was shown in supplementary figure 2.

**Ethyl sodioformyl-****β-ethoxypropionate (1).** 200ml anhydrous ether and 12g sodium wire were put into a 500ml three-necked flask. A mixture of ethyl β-ethoxypropionate (73g, 0.5mol) and ethyl formate (40g, 0.54mol) was dropped slowly into the flask during 8 hours. The reaction liquid was stirred under room temperature (RT) to form solids, and the color turned from colorless to deep yellow. Proceed to next step immediately under the protection of nitrogen.

**2-Methyl-5-ethoxymethyl-6-oxypyrimidine (2).** Under the protection of nitrogen, 12g sodium wire was put into 200ml anhydrous alcohol, heated to reflux until the sodium wire was reacted completely for producing the solution of sodium ethoxide. Acetamidine hydrochloride (45g, 0.48mol) dissolved in 100ml anhydrous alcohol was dropped slowly into the mixture of sodium ethoxide solution and the solution of compound **(1)**. The mixture solution was stirred and heated to 80^o^C to reflux overnight. Adjust the pH value of the mixture to 6 by acetic acid after cooling, then remove solvent by rotary evaporation. 50ml H_2_O was added to the residue and then extracted it twice with 100ml dichloromethane (DCM). The organic phase was dried over anhydrous sodium sulfate and purified by chromatography on a silica gel column (methanol : DCM, 1 : 100) to give 20.1g compound **(2)** as light yellow solids. The yield was 24%. ^1^H NMR (300 MHz, CDCl_3_) *δ*: 8.02 (s, 1H), 4.40 (s, 2H), 3.62 (q, 2H, *J*=6.9 Hz), 2.48 (s, 3H), 1.26 (t, 3H, *J*=7.1 Hz). MS (ESI): *m*/*z* 169 (M+1, 100).

**2-Methyl-5-ethoxymethyl-6-chloropyrimidine (3)**. 16ml phosphorus oxychloride (POCl_3_) was poured into a 100ml one-necked flask containing compound **(2)** (2g, 12mmol). The flask was heated at 78^o^C and dropped 0.4ml trimethylamine (TEA). 3 hours later, TLC analysis showed no starting material present (supplementary figure 3A). The reaction mixture was cooled to RT and the excess POCl_3_ was removed via rotary evaporation. 10ml H_2_O was added into the flask, followed by neutralizing the pH value using NaHCO_3_. The solution was extracted with 50ml DCM for three times, then the organic phase was dried via rotary evaporation. The residue was purified by chromatography on a silica gel column (methanol : DCM, 1 : 100) to give 1.7g compound **(3)** as yellow oil. The yield was 77%. ^1^H NMR (300 MHz, CDCl_3_) *δ*: 8.64 (s, 1H), 4.56 (s, 2H), 3.64 (q, 2H, *J*=7.1 Hz), 2.71 (s, 3H), 1.29 (t, 3H, *J*=6.9 Hz). MS (ESI): *m*/*z* 187 (M+1, 100).

**2-Methyl-5-ethoxymethyl-6-aminopyrimidine (4).** Compound **(3)** (1.5g, 8.1mmol) was added to a solution of ammonia (7.1M) in methanol (39ml). The mixture was heated to 140^o^C for 4.5 hours until TLC analysis showed no starting material existed (supplementary figure 3A). The reaction solution was cooled and rotary evaporated. Residue was purified via dry column chromatography (methanol : DCM, 1 : 100) to give 1.1g compound **(4)** as white solids. The yield was 82%. ^1^H NMR (300 MHz, CDCl_3_) *δ*: 7.99 (s, 1H), 5.47 (s, 2H), 4.43 (s, 2H), 3.49 (q, 2H, *J* =7.1 Hz), 2.50 (s, 3H), 1.23 (t, 3H, *J* =7.1 Hz). MS (ESI): *m*/*z* 168 (M+1, 100).

**2-Methyl-5-bromomethyl-6–aminopyrimidine Hydrobromide (5).** Compound **(4)** (1.1g, 6.6mmol) was added into a 200ml flask, followed by adding 73ml of glacial acetic acid containing 10% hydrobromic acid. The solution was heated to 100^o^C for 2 hours, then cooled to RT. The solution was filtered under vacuum, and the residue was washed by ether, reprecipitated twice by methanol/anhydrous ether to give 1.5g compound **(5)** as white solids. The yield was 81%. ^1^H NMR (300 MHz, DMSO-*d_6_*) *δ*: 9.31 (s, 1H), 8.62 (s, 1H), 8.50 (s, 1H), 4.67 (s, 2H), 2.49 (s, 3H). MS (ESI): *m*/*z* 202 (M+1, 20), 154 (M-Br+OCH_3_, 100).

**2-(4-methylthiazol-5-yl)ethyl 4-methylbenzenesulfonate (6).** Under the protection of nitrogen, 4-methyl-5(beta-hydroxyethyl)-thiazole (2.9g, 20mmol), Tosyl chloride (19.1g, 100mmol), and TEA (6.07g, 60mmol) were stirred with 100ml anhydrous DCM at RT for 16 hours until TLC analysis showed no starting material existed (supplementary figure 3A). Solvent was removed via rotary evaporation, and residue was purified by column chromatography twice (1^st^: DCM : methanol, 100 : 1. 2^nd^: DCM : methanol, 80 : 1) to give 3.2g compound **(6)**. The yield was 71%. ^1^H NMR (300 MHz, CDCl_3_) *δ*: 8.56 (s, 1H), 7.72 (d, 2H, *J* ＝ 6.6 Hz), 7.31 (d, 2H, *J* ＝ 8.1 Hz), 4.17 (t, 2H, *J* ＝ 6.6 Hz), 3.12 (t, 2H, *J* ＝ 6.6 Hz), 2.45 (s, 3H), 2.33 (s, 3H). MS (ESI): *m*/*z* 298 (M+1, 100).

**5-(2-Fluoroethyl)-4-methylthiazole (7).** Under the protection of nitrogen, Compound **(6)** (200 mg, 0.67 mmol), KF (377 mg, 6.5 mmol)，K_222_ (254 mg, 0.67 mmol), and 5ml anhydrous acetonitrile (MeCN) was added into a 25ml flask and heated at 90^o^C for 5 hours. the TLC analysis result was showed in supplementary figure 3A. The reaction solution was cooled and the solvent was removed via rotary evaporation. The product was purified by column chromatography (DCM : methanol, 200 : 1) to give 50mg compound **(7)** as yellow oil. The yield was 51%.

**3-[(4-Amino-2-methylpyrimidin-5-yl)methyl]-5-(2-fluoroethyl)-4-methylthiazolium Bromide Hydrobromide (cold standard sample of ^18^F-deoxy-thiamine).** Compound **(7)** (255 mg, 1.76 mmol) and **(5)** (441 mg, 1.57 mmol) were dissolved into 3 ml anhydrous MeCN and heated to 110^o^C for 20 min. 5ml hot alcohol was added into the reaction solution, and the solution was cooled to RT and filtered under vacuum to give 550mg crude product that was reprecipitated by methanol/ether to give 500mg purified cold standard sample of ^18^F-deoxy-thiamine. The yield was 75%. ^1^H NMR (300 MHz, DMSO-*d_6_*) *δ*: 9.85 (s, 1H), 8.32 (s, 1H), 5.46 (s, 2H), 4.78 (t, 1H, *J* =5.4 Hz), 4.63 (t, 1H, *J*=5.4 Hz), 3.46 (t, 1H, *J=*5.4 Hz), 3.37 (t, 1H, *J=*5.4 Hz), 2.57 (s, 3H), 2.53 (s, 3H). MS (ESI): *m*/*z* 267 (M+1, 100).

**2. The details of synthesis and purification for intermediate product [^18^F]-compound (7) and ^18^F-deoxy-thiamine**

110-120 GBq (3-3.24 Ci) ^18^F^-^ ions were produced by the cyclotron and transferred by argon gas stream into the QMA column. Then the ^18^F^-^ ions were eluted into reaction vial I by the eluent in A1. After adding 1 ml MeCN from A2 into vial I, the mixture was heated at 120^o^C for 3 min, then argon gas stream was blown into vial I for 3 min. Vial I was cooled to room temperature (RT), then added another 1 ml MeCN from A4, heated at 120^o^C for 3 min with argon gas stream blown. Then, the H_2_O component of the eluent was distilled by azeotrope completely. After adding a solution of 5 mg precursor **(6)** in 0.5 ml MeCN from A3, vial I was sealed and incubated at 110^o^C for 20 min for synthesizing the intermidiate product [^18^F]-compound **(7)**.

Vial I was cooled to RT for stablizing the reaction mixture, then heated to 120^o^C in order to distill [^18^F]-compound **(7)** and the solvent MeCN from vial I to vial II through a connecting tube. Argon gas stream was started for assisting the transfer of [^18^F]-compound **(7)** and solvent. The middle part of the connecting tube was curved and immersed in ice/water bath, in order to condense [^18^F]-compound **(7)** and solvent before arriving at vial II. Meanwhile, vial II was cooled to 4^o^C for further condensing the arrived sample. When the distillation-transfer process lasted for 2 mins, vial I was cooled to RT, added 0.5ml MeCN from A5 for rinse, re-heated to 120^o^C to continue. The whole distillation-transfer process lasted for 6.5 mins, then vial II was sealed and heated to 110^o^C for 20 min for synthesizing the final product ^18^F-deoxy-thiamine. 5mg powder of precursor **(5)** had been added in vial II beforehand.

After Cooling to RT for stabilizing raction mixture, solvent MeCN was dried by heating vial II to 110^o^C for 3 min with argon gas stream blown. Then, 0.5 ml H_2_O from B1 was added into vial II for dissolving hydrophilic ^18^F-deoxy-thiamine. The solution mixture was injected into prep-HPLC system installed on RNplus module for purification. A semi-preparation column (250 * 10 mm, 5 um; Luna, Phenomenex) was used and the mobile phase was water containing 5 mM sodium acetate and 0.05% glacial acetic acid. The flow rate was 5 ml/min, and the retention time of ^18^F-deoxy-thiamine was around 10 min. The mobile phase containing ^18^F-deoxy-thiamine was collected into collection vial 1 for half a minute, then transferred to sterile collection vial 2 through a 0.2 um sterile filter (Millipore). The whole automated synthesis duration was 100 mins.

**3. The details of Patlak and Logan models:**

Patlak model:

$\frac{C_{T}(t)}{C_{p}(t)}=K_{i}\frac{\int_{0}^{t} C_{p}(\tau)d\tau}{C_{p}(t)}+V_{0}$,

where *t* represents time after ^18^F-deoxy-thiamine injection, C_T_(*t*) is the SUV of ^18^F-deoxy-thiamine in whole brain at time *t*, C_P_(*t*) is the SUV of blood at time *t*, K_i_ is the transfer constant of ^18^F-deoxy-thiamine entering brain when steady state reached. *∫*_0_^t^C_p_($\tau$)d$\tau$ represents the AUC of the TAC in blood from time 0 to time t after ^18^F-deoxy-thiamine injection. V_0_ is the distribution volume of ^18^F-deoxy-thiamine at time 0, no practical meaning in the model.

Logan model:

$\frac{\int_{0}^{t} C_{T}(\tau)d\tau}{C_{T}(t)}=V_{D}\frac{\int_{0}^{t} C_{p}(\tau)d\tau}{C_{T}(t)}+int$,

where *t* represents time after ^18^F-deoxy-thiamine injection, C_T_(*t*) is the SUV of ^18^F-deoxy-thiamine in liver or kidney at time *t*, *∫*_0_^t^C_T_($\tau$)d$\tau$ represents the AUC of the TAC in liver or kidney from time 0 to time *t* after ^18^F-deoxy-thiamine injection. *∫*_0_^t^C_p_($\tau$)d$\tau$ represents the AUC of the TAC in blood from time 0 to time *t* after ^18^F-deoxy-thiamine injection. V_D_ is the distribution volume of ^18^F-deoxy-thiamine in liver or kidney when steady state reached.

**Supplementary table 1. the renal excretion rate of ^18^F-deoxy-thiamine 85 mins after tail vein injection**

| ICR mice | injected radioactivity (MBq) | renal excreted radioactivity 85 mins after injection (MBq) | excretion rate (%，decay-correction to injection time) |
| --- | --- | --- | --- |
| 1 | 7.74 | 2.79 | 35.99 |
| 2 | 13.71 | 3.67 | 26.78 |
| 3 | 16.11 | 6.39 | 39.70 |

**Supplementary table 2. biodistribution of ^18^F-deoxy-thiamine in ICR male mice**

| organs or tissues | biodistribution (IA%/g, mean±SEM) | | | | | |
| --- | --- | --- | --- | --- | --- | --- |
|  | 5 min | 15 min | 30 min | 60 min | 120 min | 240 min |
| kidney | 12.61±2.42**^a^** | 10.94±2.26 | 3.62±0.24 | 1.13±0.20 | 0.63±0.05 | 0.19±0.03 |
| liver | 13.86±4.17 | 8.24±0.60 | 2.58±0.31 | 1.06±0.10 | 0.97±0.06 | 0.32±0.02 |
| duodenum | 17.98±5.99 | 11.24±0.49 | 6.34±1.02 | 1.75±0.23 | 2.22±0.74 | 1.94±0.41 |
| pancreas | 6.92±2.62 | 5.55±0.48 | 1.93±0.46 | 0.66±0.06 | 0.47±0.03 | 0.44±0.12 |
| lung | 3.96±0.79**^a^** | 7.05±1.41 | 2.47±1.49 | 1.23±0.20 | 0.75±0.08 | 1.18±0.43 |
| fumur | 5.60±2.73 | 2.95±0.44 | 3.15±0.16 | 3.98±0.94 | 3.03±0.07 | 1.82±0.22 |
| stomach | 5.48±2.05 | 6.68±1.47 | 2.78±0.87 | 1.52±0.23 | 1.35±0.33 | 0.97±0.12 |
| heart | 2.31±0.55 | 2.17±0.46 | 0.84±0.14 | 0.44±0.08 | 0.23±0.02 | 0.16±0.02 |
| blood | 2.39±0.60 | 1.53±0.36 | 0.86±0.05 | 0.39±0.03 | 0.30±0.01 | 0.12±0.004 |
| spleen | 2.40±0.56 | 3.52±0.43 | 1.44±0.23 | 0.66±0.10 | 0.47±0.05 | 0.24±0.02 |
| brain | 1.38±0.46 | 1.51±0.16 | 0.88±0.15 | 0.48±0.05 | 0.41±0.06 | 0.10±0.005 |
| muscle | 1.43±0.12 | 1.89±0.16 | 1.68±0.42 | 2.21±0.79 | 0.82±0.08 | 0.47±0.10 |
| fat | 1.67±0.61 | 1.61±0.93 | 0.49±0.11 | 0.44±0.08 | 0.40±0.05 | 0.29±0.08 |
| testicle | 1.23±0.31 | 1.24±0.13 | 0.87±0.05 | 0.32±0.08 | 0.58±0.07 | 0.23±0.01 |

Note: Radioactivity accumulation was expressed as %IA/g at 5, 15, 30, 60, 120, 240 min after tail-vein injection of 3.7 MBq ^18^F-deoxy-thiamine in 0.1 ml volume (n=3 for each organ or tissue at eact time point).

**a.** n=2 for kidney and lung at 5min.

**Supplementary table 3. biodistribution of ^18^F-deoxy-thiamine in ICR female mice**

| organs or tissues | biodistribution (IA%/g, mean±SEM) | | | | | |
| --- | --- | --- | --- | --- | --- | --- |
|  | 5 min | 15 min | 30 min | 60 min | 120 min | 240 min |
| kidney | 42.35±2.82**^a^** | 8.47±1.56 | 6.18±1.18 | 2.09±0.24 | 1.67±0.63 | 0.50±0.22 |
| liver | 17.60±6.41**^a^** | 8.33±1.96 | 4.09±0.82 | 2.00±0.07 | 1.59±0.49 | 0.42±0.08 |
| duodenum | 23.89±14.71**^a^** | 10.23±3.09 | 8.73±3.97 | 6.44±1.61 | 2.90±0.31 | 2.01±0.37 |
| pancreas | 10.46±3.61 | 3.86±0.91 | 2.58±0.77 | 1.22±0.09 | 0.83±0.26 | 0.33±0.05 |
| lung | 9.33±3.12**^a^** | 3.34±0.93 | 2.76±0.33 | 1.89±0.10 | 1.31±0.39 | 0.58±0.09 |
| fumur | 4.49±1.07 | 2.97±0.82 | 2.68±0.80 | 4.82±0.79 | 2.16±0.37 | 2.06±0.07 |
| stomach | 7.68±2.27 | 3.36±0.67 | 2.24±0.43 | 2.33±0.38 | 1.37±0.10 | 0.45±0.09 |
| heart | 3.98±0.92 | 1.46±0.36 | 0.89±0.18 | 0.48±0.01 | 0.36±0.07 | 0.16±0.02 |
| blood | 3.87±0.51 | 1.59±0.42 | 0.89±0.12 | 0.52±0.05 | 0.39±0.07 | 0.16±0.01 |
| spleen | 4.90±2.28 | 2.62±0.53 | 2.08±0.51 | 1.26±0.12 | 0.56±0.30 | 0.22±0.03 |
| brain | 1.62±0.35 | 1.18±0.29 | 0.97±0.24 | 0.72±0.06 | 0.52±0.06 | 0.14±0.01 |
| muscle | 4.80±1.53 | 1.92±0.28 | 1.55±0.47 | 1.43±0.22 | 0.80±0.17 | 0.71±0.28 |
| fat | 1.98±0.73 | 0.97±0.04 | 0.44±0.07 | 0.35±0.03 | 0.24±0.02 | 0.21±0.03 |
| ovary | 4.92±1.44**^a^** | 2.78±0.52 | 2.88±1.26 | 1.29±0.19 | 0.84±0.28 | 0.30±0.04 |

Note: Radioactivity accumulation was expressed as %IA/g at 5, 15, 30, 60, 120, 240 min after tail-vein injection of 3.7 MBq ^18^F-deoxy-thiamine in 0.1 ml volume (n=3 for each organ or tissue at eact time point).

**a.** n=2 for kidney, liver, duodenum, lung, and ovary at 5min.
